# *Actinomucor elegans* and *Podospora bulbillosa* Positively Improves Endurance to Water Deficit and Salinity Stresses in Tomato Plants

**DOI:** 10.3390/jof8080785

**Published:** 2022-07-27

**Authors:** Elham Ahmed Kazerooni, Sajeewa S. N. Maharachchikumbura, Abdullah Mohammed Al-Sadi, Umer Rashid, Sang-Mo Kang, In-Jung Lee

**Affiliations:** 1 Department of Applied Biosciences, Kyungpook National University, Daegu 41566, Korea; elham.ghasemi.k@gmail.com (E.A.K.); rhizobacteria@gmail.com (S.-M.K.); 2 School of Life Science and Technology, Center for Informational Biology, University of Electronic Science and Technology of China, Chengdu 611731, China; sajeewa83@yahoo.com; 3 Department of Plant Sciences, College of Agricultural and Marine Sciences, Sultan Qaboos University, P.O. Box 34, Al-Khod 123, Oman; alsadi@squ.edu.om; 4 Institute of Nanoscience and Nanotechnology (ION2), Universiti Putra Malaysia, Serdang 43400, Selangor, Malaysia; dr.umer.rashid@gmail.com

**Keywords:** lipid metabolism, hydrogen peroxide, amino acid, antioxidant enzymes, sugar, phytohormone, salicylic acid

## Abstract

Fungal strains isolated from the rhizosphere of healthy *Solanum lycopersicum* were examined to mitigate symptoms of drought and salinity stresses. The fungal strains were identified as *Actinomucor elegans* and *Podospora bulbillosa* based on their DNA sequencing and morphological analysis. Additionally, the fungal strains were assayed for a number of plant growth promoting traits and abiotic stresses on solid media. Moreover, a greenhouse experiment was conducted and tomato seedlings were treated with 25% PEG or 1.5% NaCl for 12 days, and the impact of plant growth promoting fungi (PGPF) on tomato seedling performance under these conditions was examined. PGPF application raised the survival of the stressed tomato plants, which was evidenced by higher physiological and biochemical processes. The PGPF-inoculated plants exhibited higher chlorophyll, carotenoid, protein, amino acid, antioxidant activities, salicylic acid, glucose, fructose, and sucrose contents, and showed lower hydrogen peroxide, and lipid metabolism relative to control plants under stress. Analysis using gene expression showed enhanced expression of *SlF3H* gene and reduced expression of *SlNCED1*, *SlDEAD31*, *SlbZIP38*, and *SlGRAS10* genes following PGPFs application. Overall, the outcomes of this study elucidate the function of these fungal strains and present candidates with potential implementation as biofertilizers and in promoting plant stress endurance.

## 1. Introduction

Plants, as sessile organisms in nature, are continuously exposed to myriads of environmental stresses. As a consequence, plants have evolved arrays of adaptive mechanisms such as hormone regulation, changing morphological structure, osmotic regulation, and scavenging mechanisms to acclimatize to detrimental conditions [1]. Environmental stresses in the shape of extreme temperature, salinity, chill, flood, and water deficit severely impair plant performance and development and restrain plant production [2,3,4,5]. Salinity is a devastating ecological and agronomical matter, which has been magnified by the intensive salinization of lands [6]. Variable intensities of salt cause photosynthetic inhibition, nutrient disorder, oxidative injury, membrane incompetence, enzyme damage, premature senescence, and metabolic toxicity [7,8], which interferes with all growth stages, comprising seed germination, seedling formation, and fertility ratio [9]. Drought, known as water stress, is one of the serious challenges to crop production around the world [7]. It has been anticipated that water stress prevalence and intensity will surge in diverse zones in the future due to global climate change, deforestation, and urbanization [10,11]. This abiotic stress negatively influences the world’s food security, and its impact has rapidly been accentuated following the decreased approachability of surface water resources [12,13].

The rhizosphere region around plants’ roots has a high diversity of microorganisms including beneficial and pathogens [14]. Plant growth-promoting fungi (PGPF) are a diverse group of nonpathogenic fungi that are associated with plants and have beneficial effects on plants [15]. They usually colonize the root system to improve growth, development, and protection [16]. Extensive research data have shown that PGPF can produce plant growth regulators, solubilize phosphate, mobilize nutrients, solubilize metal, and produce organic acid and extracellular enzymes [17,18,19]. Moreover, PGPF has the ability to detoxify and fasten the degradation of toxic compounds in soils that have harmful effects on plants [19] and confer plant tolerance to harsh environmental conditions such as salinity, drought, and flooding [20]. 

Tomato (*Solanum lycopersicum* L.), is one of the top-produced vegetables with high economic value in the world [21]. Tomato is a great source of phytochemicals and nutrients including vitamins, minerals, and antioxidants, which have been connected to numerous health benefits [22]. However, tomato is not classified as a resistant plant in reaction to many abiotic stresses and its production has been drastically reduced by adverse environmental conditions, in particular drought and salinity [23]. 

*Actinomucor elegans* (order *Mucorales*), a fast-growing and enzyme-producing fungus, was earlier isolated from soil samples [24]. This fungal species is known for its connection with the production of soy-based products, providing flavor and texture to food [25]. Similarly, *Podospora bulbillosa* belongs to the order *Sordariales*. Fungi belonging to this order are a famous source of secondary metabolites with potential beneficial applications. They are commonly found in soil and other habitats such as wood, animal dung, or herbaceous plants [26]. Despite some knowledge available about these fungal species, no reports exist on their ability to mitigate salinity and drought stress in tomatoes or other plants.

Due to the fact that climate change has increased the frequency and intensity of varied abiotic stresses, it is crucial to provide crops with multiple stress endurance to reduce the pressure of environmental changes and fulfill the demand of the rapid growth of the human population. It was hypothesized that *Actinomucor elegans* and *Podospora bulbillosa* could promote water deficit and salinity endurance in tomato. Hence, this study was conducted to investigate whether the application of *A. elegans* and *P. bulbillosa* could improve recovery ability and support the growth of tomatoes under selected abiotic stresses. Specific objectives of the study include: (1) to investigate the ability of *A. elegans* and *P. bulbillosa* to support tomato growth under salinity and drought stresses; (2) to assess the impacts of *A. elegans* and *P. bulbillosa* applications on varied physiochemical traits such as agronomic attributes, antioxidant operations, amino acid contents, and phytohormone content in drought and salinity-stressed plants; (3) to clarify the regulatory functions of *A. elegans* and *P. bulbillosa* in tomato plants under normal growth/stress conditions; and (4) to authenticate our physiochemical results through the expression patterns of varied stress-responsive genes including *SlF3H*, *SlNCED1*, *SlDEAD31*, *SlbZIP38*, and *SlGRAS10*. 

## 2. Materials and Methods

### 2.1. Sampling of Rhizosphere Soil and Isolation of Rhizospheric Fungi 

Soil samples were collected from the rhizosphere zone of tomato plants grown on an agricultural farm located in Gunwi-gun district (36°06′48.5″ N 128°38′26.4″ E) (Daegu, South Korea). Samples were collected from randomly chosen healthy tomato plants. The collected samples, comprising roots and root-adhered soil, were subjected to serial dilution for the isolation of fungi following a method described previously [27,28]. Root samples were vortexed at high speed (90 s) to remove adhered soil from the root. The collected adhered soil was subjected to serial dilution. Moreover, root samples were vortexed in sterile distilled water (2 times) and surface-sterilized with 1.6% sodium hypochlorite (16 min) and 95% ethanol (1 min), followed by three times washing with sterile distilled water. After that, root samples were macerated in sterile distilled water using a sterile pestle and mortar, diluted and 100 μL of it was added to the prepared media and incubated at 27 °C for 7 days. Finally, the pure cultures of isolated fungi were preserved on potato dextrose agar (PDA) slants and used for further studies.

### 2.2. Evaluation of Fungal Strains for Plant-Growth Promotion Activities 

Selected fungal strains were screened for some plant growth-promoting properties. Production of indole-3-Acetic Acid (IAA) by fungal strains was evaluated by growing the strains in a medium amended with/without L-tryptophan (5 mM) [29]. After incubation (48 h, 27 °C), Salkowski’s reagent (4 mL) was added to supernatant (1 mL) and incubated in the darkness for 30 min. The development of pink to red color demonstrates IAA production by fungal strains. Chrome azurol S (CAS) agar medium was used to examine siderophore production by the fungal strains [30]. The reaction was contemplated positive when a yellow-orange halo surrounding the fungal colony emerged as a result of the iron removal from CAS by the siderophore. To examine ammonia production, the fungal strains were grown in peptone broth for 48–72 h at 27 °C. Afterward, Nessler’s reagent was added to the fungal suspension and the emergence of yellow to brown color confirmed ammonia production [29]. 

### 2.3. Assessment of Fungal Strains for Stress Tolerance under Laboratory Condition 

A diverse range of abiotic stress tolerance tests (salinity, drought, and pH) was performed on different levels with a fresh culture of the isolated fungi. The unaltered medium served as a control. Salinity tolerance stress was checked by growing the fungal strains on the PDA media, which was amended with diverse concentrations (0.5%; 0.086 M, 2.5%; 0.43 M, 5%; 0.86 M, 7.5%; 1.29 M, and 10%; 1.72 M *w*/*v*) of sodium chloride (NaCl). The plates were incubated for 7 d at 25 ± 2 °C. Tolerance to drought stress was examined by observing the growth of fungal strains on PDA plates supplemented with various osmotic potentials (−0.05, −0.15, −0.3, −0.49, and −0.73 MPa) of polyethylene glycol (PEG 6000 Da) [31]. The pH resistance of fungal strains was evaluated on a PDA medium amended with various pH regimes (2, 5, 8, and 12) at 25 °C for 7 d. 

### 2.4. Molecular Identification and Phylogenetic Analysis of the Fungal Strains

Genomic DNA was extracted from fresh fungal cultures (seven days old) following the protocol described previously [32]. Molecular identification was performed on the basis of fungal internal transcribed spacer (ITS) rDNA for *Actinomucor elegans* and (ITS) rDNA + LSU for *Podospora bulbillosa*. Amplification of fungal ITS and LSU regions was conducted using the BioFACT™ 2X Multi-Star PCR Master Mix (BIOFACT, Daejeon, Korea) and a combination of ITS1/ITS4 and LR7/LR0R, respectively [33,34,35]. PCR products were examined for the expected size on 1% agarose gel and sequenced by SolGent Co., Ltd. (Daejeon, Korea). The sequences of the fungal strains (PGPF1 and PGPF2) reported in this study were submitted to GenBank. Phylogenetic analyses were implemented using the RAxML GUI v.1.3 [36] and a Phylogenetic tree was constructed using FigTree v. 1.4.0 (http://tree.bio.ed.ac.uk/software/figtree/, accessed on 11 May 2022). 

### 2.5. Effect of the Fungal Strains on Tomato Growth under Drought, and Salinity

Seeds of tomato, *Solanum lycopersicum* (rendered by Danong Co., Ltd., Namyangju, South Korea) were surface-sterilized with 70% ethanol (3 min) and 1% sodium hypochlorite (10 min), followed by four times washing with sterile distilled water. Seeds were sown in pot trays containing sterilized horticultural soil (pH ~ 7, and EC ≤ 1.2) (Shinsung Mineral Co., Ltd., Daegu, Chungcheongbuk-do, Republic of Korea). Soil sterilization was carried out by autoclaving at 15 lbs/121 °C for 3 h. Seedlings were pre-grown under standard greenhouse conditions (24 ± 2 °C, 70% relative humidity, and 14 h/10 h light/dark regime). When the germinated seeds had grown for three weeks, tomato seedlings with uniform growth were used to perform varied treatments (Table 1). One plant per pot was allowed and transplanted to a pot containing sterilized soil. Before stress treatments, they were divided into three groups: control, PGPF1, PGPF2, and all groups were treated equally (50 mL/pot). The control group was irrigated daily only with sterile distilled water, while the PGPF1 and PGPF2 groups were irrigated with chosen fungal suspensions for 12 days. Briefly, the inoculum suspension was prepared from 7-day-old cultures of the fungal strains based on a previously described method by Zhang, et al. [37]. The spore density was examined with a hemocytometer, and the final suspension (1.0 × 10^8^ spore/mL) was maintained at 4 °C. Then, PGPF treated and untreated seedlings were separated into different groups and received various treatments including 1.5% sodium chloride (1.5% NaCl) and 25% polyethylene glycol 6000 (25% PEG6000; −0.73 Mpa) for 12 days (50 mL/pot). In each case, tomato seedlings were tested for each treatment in five replicates. Eventually, tomato leaves (third or fourth leaf from the growing tip) were taken from 47-day-old seedlings (between 9:30 and 11:00 a.m.), and flash-frozen in liquid nitrogen prior to maintaining at −80 °C. 

### 2.6. Growth Parameter and Photosynthetic Pigment Assessment 

At the completion of the treatment period, varied agronomic traits were measured to investigate the influence of each treatment on the seedlings. The following measurements consisted of plant height, root length, stem diameter, leaf area, plant fresh/dry weight, and root fresh/dry weight, which were recorded at 12 days post-treatment and compared with control seedlings. Plant height and root length were measured using a ruler. A digital Vernier caliper was used to measure stem diameter, leaf length, and leaf width. Aboveground parts and roots were thoroughly cleaned and oven-dried at 60 °C for 48 h to determine their dry weights. 

Chlorophyll a (Chl a), chlorophyll b (Chl b), and carotenoid were estimated in tomato leaves using a method by Bharti, et al. [38], Pan, et al. [39]. Photosynthetic pigments were extracted from fresh tomato leaves with 80% acetone via centrifugation (10,000 rpm, 10 min). The spectral absorbance of the supernatant was determined at 663, 646, and 470 nm with a spectrophotometer (Thermo Fisher Scientific, Waltham, MA, USA).

### 2.7. Estimation of Salicylic Acid (SA) Content

Briefly, SA extraction was carried out by adding 80% ice-cold methanol (0.5 mL) to a freeze-dried sample (0.1 g) and shaking at 4 °C for 30 min [40]. Afterward, the solution was centrifuged (12,000 rpm, 30 min, 4 °C) and the collected supernatant was used for SA quantification via high-performance liquid chromatography (HPLC).

### 2.8. Examination of Amino Acid Composition

Then, 50 mg of freeze-dried sample were macerated with an acidic mixture comprising 2 mL hydrochloric acid (6 M HCl), 1 mL anhydrous trifluoroacetic acid (TFA), and 0.5% 2-mercaptoethanol (2-ME). Hydrolysis was done for 40 min at 170 °C under N_2_ in sealed ampules [41]. The obtained hydrolysate was dried out with an evaporator, dissolved in distilled water (1 mL), and centrifuged (12,000 rpm, 2 min, 4 °C). The amino acid composition was evaluated with an Amino Acid analyzer (Hitachi High-Technologies Corporation, Tokyo, Japan). 

### 2.9. Determination of Soluble Protein and Sugar Concentrations 

Estimation of protein concentration in tomato leaves was carried out according to the described procedure of Bradford [42], following minor modification as indicated by Kazerooni, et al. [43]. The leaf tissues were homogenized in 80% (*v*/*v*) ethanol and the extraction procedure for soluble sugar was carried out following the protocol of Zhu, et al. [44]. The obtained extraction was dried and the dehydrated remnant was dissolved in 50% acetonitrile and preserved at −20 °C. The extracts were passed through a filter (0.45 µm) before HPLC (high-performance liquid chromatography) injection (Millipore Co., Waters Chromatography, Milford, MA, USA).

### 2.10. Enzyme Assay in Tomato Plants

The superoxide dismutase (SOD) and catalase (CAT) enzymes were extracted by the defined method by Du, et al. [45]. Polyphenol oxidase (PPO) and peroxidase (POD) contents were ascertained in plant leaves using Catechol (50 mM) and pyrogallol (50 mM), respectively, based on the protocol proposed by Soffan, et al. [46]. The procedure of Zhou, et al. [47] was used for the determination of flavonoids and DPPH (2,2-diphenyl-1-picrylhydrazyl) activity. The absorbance of the resulting mixtures was read at specific wavelengths by a spectrophotometer (Thermo Fisher Scientific, Waltham, MA, USA).

### 2.11. Detection of Hydrogen Peroxide (H_2_O_2_) and Malondialdehyde in Tomato Plants

The method described by Batista-Silva, et al. [48] was followed to measure hydrogen peroxide content (H_2_O_2_) in plant extracts. Briefly, freeze-dried plant samples (30 mg) were dissolved in 1 mL of 0.6% TBA (thiobarbituric acid) in 10% TCA (trichloroacetic acid). Afterward, the mixture was heated (100 °C, 15 min) and centrifuged (12,000 rpm, 20 min). Then, the supernatant (20 µL) was mixed with 100 mM phosphate buffer (200 µL) and 1% potassium iodide (80 µL). Eventually, the mixture absorbance was recorded at 390 nm by a spectrophotometer (Thermo Fisher Scientific, Waltham, MA, USA).

The amount of lipid peroxidation was estimated by referring to the malondialdehyde (MDA) content in tomato plants. Leaf samples (0.2 g) of each treatment were homogenized in 1.0% TCA (4 mL) and centrifuged at room temperature (12,000 rpm, 5 min). Then, 1 mL of supernatant was added to 20% TCA (4 mL) containing 0.5% TBA (thiobarbituric acid). The mixture was heated using boiling water (95 °C, 30 min), and then cooled in an ice bath (10 min). Subsequently, the mixture was centrifuged (12,000 rpm, 10 min) and the supernatant absorbance was recorded at 532 and 600 nm with a spectrophotometer (Thermo Fisher Scientific, Waltham, MA, USA). 

### 2.12. Quantification of Nutrient Content in Tomato Plants and Fungal Mycelia

Plants grown under varied treatments were freeze-dried and processed into powder to determine their nutrient contents. Plant samples were prepared based on the described method by Choi, et al. [49]. Briefly, the powdered sample was digested in nitric acid (0.6 mL) in a glass test tube at 120 °C for 2 h followed by additional digestion in 60% HClO_4_ (0.4 mL, 150–180 °C, 2 h). The resulting sample was cooled to room temperature and diluted in Nanopure water (5 mL). Moreover, fungal strains were grown in potato dextrose broth containing 1.5% sodium chloride (1.5% NaCl) and 25% polyethylene glycol 6000 (25% PEG6000). After that, the obtained fungal mycelia were processed for nutrient analysis [50]. Eventually, sodium (Na), potassium (K), phosphorus (P), and calcium (Ca) contents were measured in plant and fungal mycelia with an inductively coupled plasma mass spectrometer (Optima 7900DV, Perkin-Elmer, Akron, OH, USA). 

### 2.13. RNA Isolation and Gene Expression by Quantitative Real-Time PCR

Total RNA was extracted from the tomato leaves receiving various treatments using the Trizol reagent (Sigma-Aldrich Inc., St. Louis, MO, USA) [51]. RNA quality and quantity was checked by NanoDrop 2000 spectrophotometer (Thermo Fisher Scientific, Wilmington, DE, USA). The RNA was reverse transcribed to cDNA and used for quantitative real-time PCR (qRT-PCR) assay. For each sample, 1 µg of total RNA was used to generate cDNA by employing a BioFACT RT-Kit (BIOFACT, Daejeon, Korea), and qRT-PCR was performed on an Illumina’s Eco Real-Time PCR System (llumina, San Diego, CA, USA). The relative expression of stress-related genes was detected by qRT-PCR employing the primers described in Table S1. We examined the expression patterns of varied genes including *SlF3H* [52,53], *SlNCED1* [54], *SlDEAD31* [55], *SlbZIP38* [39], and *SlGRAS10* [56]. 

### 2.14. Data Analysis

SAS statistical software (version 9.4, SAS institute, Cary, NC, USA) was employed to evaluate the data via ONE-way analysis of variance (ANOVA) by examining the effect of the two fungal strains in improving growth, photosynthesis, and other parameters in tomato plants under stress conditions. Tukey’s test at *p* < 0.05 was used to define substantial contrasts among treatments. Meaningful differences are depicted by varied letters above the bars in each figure. Graphs were portrayed with Origin Pro (version 9.85, Northampton, MA, USA). The data exhibited are means of five replicates for treatments and control.

## 3. Results

### 3.1. Phylogenetic Analysis and Plant Growth-Promoting Traits of Fungal Strains

**Figure 1:**
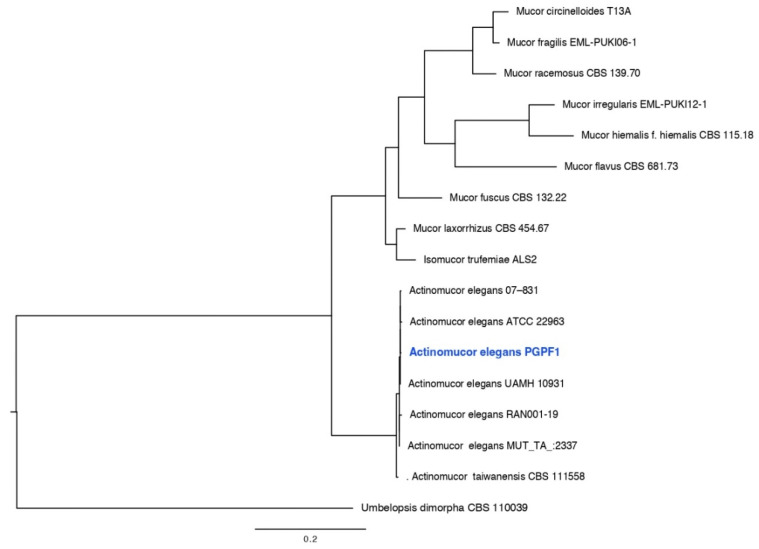
Maximum likelihood tree obtained from the ITS sequence alignment analysis of the species in section *Actinomucor elegans*. Bootstrap values (>50) are represented by numbers at the nodes based on 1000 replications.

**Figure 2:**
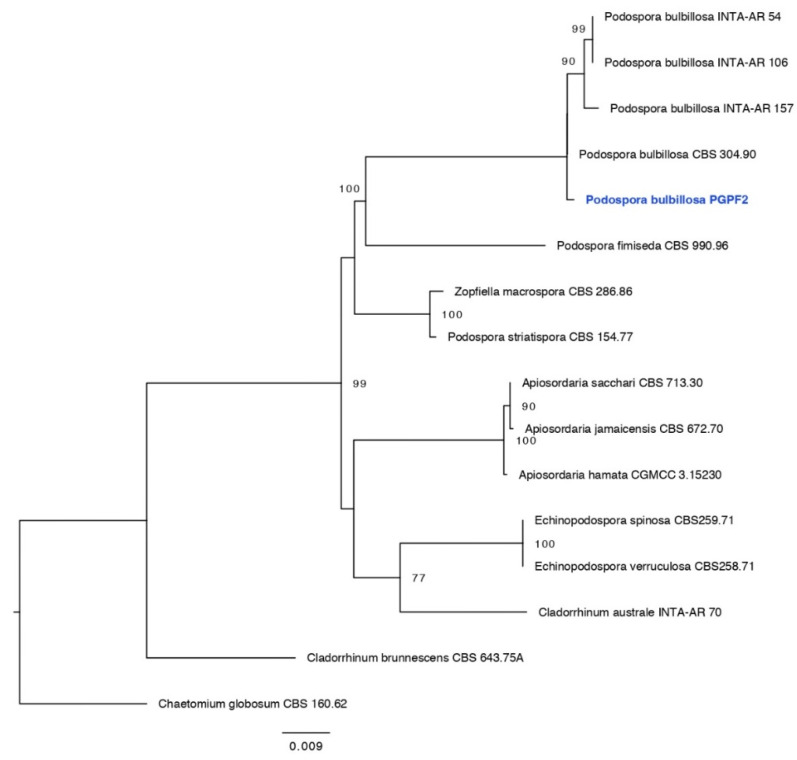
Maximum likelihood tree obtained from the ITS sequence alignment analysis of the species in section *Podospora bulbillosa*. Bootstrap values (>50) are represented by numbers at the nodes based on 1000 replications.

The ITS tree for *Actinomucor* and related taxa consisted of 17 taxa including *Umbelopsis dimorpha* (CBS 110039) as the out-group taxon. The *Actinomucor* isolate (PGPF1, GenBank accession for ITS: ON817258) obtained in this study clustered with previously published species of *Actinomucor elegans*, with 100% ML support (Figure 1). The ITS + LSU tree for *Podospora* and related taxa in Sordariaceae consisted of 15 taxa including *Chaetomium globosum* (CBS 160.62) as the out-group taxon. The *Podospora* isolate (PGPF2, GenBank accession for ITS: ON817257, LSU: ON817270) obtained in this study clustered with previously published isolates of *Podospora bulbillosa* (Figure 2).

Plant growth-promoting traits such as IAA and ammonia productions were found to be positive for both fungal strains (Figure S1). However, the fungal strains were found to be negative for siderophore production.

### 3.2. In-Vitro Resistance of Fungal Strains to Drought, Salinity, and pH 

The fungal strains were evaluated for their capabilities to endure abiotic stresses by examination of their growth under various levels of drought, salt, and pH. The fungal strain PGPF1 was proved to be resistant to all the ranges of PEG concentrations (−0.05 to −0.73 MPa) (Figure S2) while, the fungal strain PGPF2 was found to be sensitive to the PEG concentration (−0.73 MPa). The strain PGPF1 was observed to be resistant against 0.5%, 2.5% and 5% NaCl concentrations and growing slowly at 7.5% and 10% NaCl concentrations (Figure S2). Noticeable growth was detected in the presence of 0.5%, 2.5%, and 5% NaCl concentrations for the strain PGPF2, whereas at 7.5% and 10% NaCl concentrations the growth was absolutely eliminated for this strain. Thereby, we can affirm that the strain PGPF2 can contribute to tolerating salt stress to a specific extent. Furthermore, this study revealed that strain PGPF1 was able to endure pH in variable ranges (2–12 pH), However, strain PGPF2 was growing slowly at some pH ranges (2 and 12 pH) compared to strain PGPF1 (Figure S3). 

### 3.3. Tomato Plants Response to PGPF1 (A. elegans) and PGPF2 (P. bulbillosa) Inoculants under Varied Abiotic Stresses 

#### 3.3.1. Effect of PGPF on Plant Growth under Abiotic Stresses 

**Figure 3:**
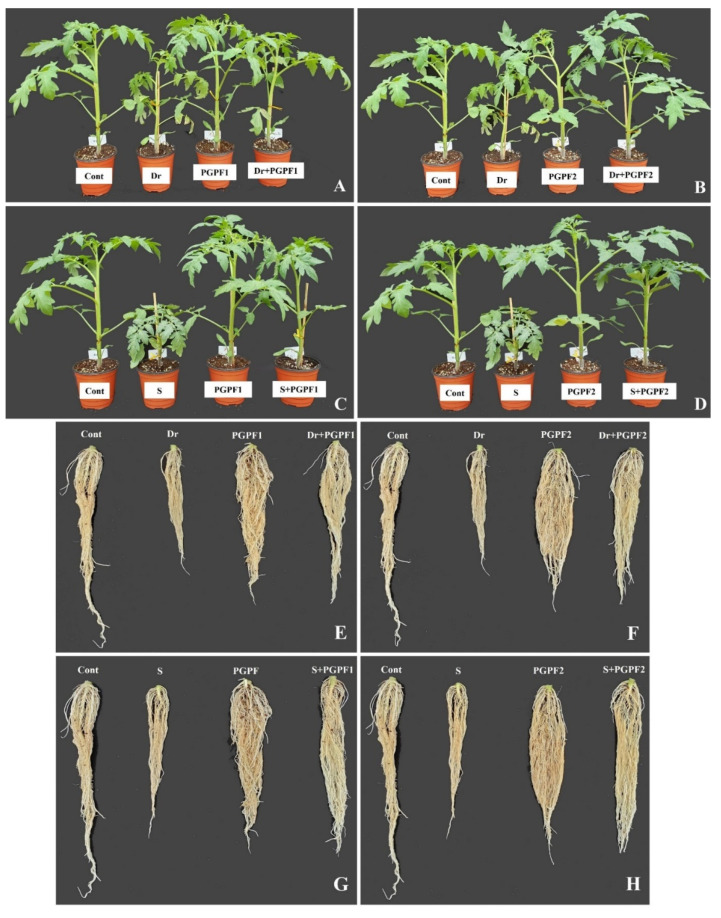
Effects of PGPF inoculation on tomato plant (**A**–**D**) and root (**E**–**H**) growth under normal and stress conditions after 12 days of treatment. Treatments: Cont (control), PGPF1 (*Actinomucor elegans*), PGPF2 (*Podospora bulbillosa*), Dr (25% Polyethylene glycol), Dr (25% Polyethylene glycol) + PGPF1 (*Actinomucor elegans*), Dr (25% Polyethylene glycol) + PGPF2 (*Podospora bulbillosa*), S (1.5 sodium chloride), S (1.5% sodium chloride) + PGPF1 (*Actinomucor elegans*), and S (1.5% sodium chloride) + PGPF2 (*Podospora bulbillosa*).

The effect of PGPF1 and PGPF2 inoculations on tomato growth was studied in both stress and non-stress environments. Drought and salinity stress caused a reduction in several growth parameters compared to unstressed and un-inoculated tomato plants (Figure 3 and Figure S4). A significant enhancement in the growth of PGPF1 and PGPF2 inoculated tomato was observed compared to un-inoculated tomato under both non-stress and stress conditions. PGPF1 and PGPF2 treated plants recorded enhanced growth parameters in terms of plant height and plant fresh weight. Plant height was improved by 24.34% (PGPF1) and 35.02% (PGPF2) under drought, and 28.66% (PGPF1) and 31.28% (PGPF2) under salt treatments, as compared to the related heights of un-inoculated stressed plants (*p* < 0.05) (Figure S4A). PGPF-inoculated plants recorded higher plant fresh weight (PGPF1, 43.64%; PGPF2, 61.19%) under drought and (PGPF1, 49.21%; PGPF2, 44.31%) under salt treatments, in contrast to the plant fresh weight of the un-inoculated stressed plants (Figure S4F).

#### 3.3.2. PGPF Modulates Photosynthetic Pigments under Abiotic Stresses

**Figure 4:**
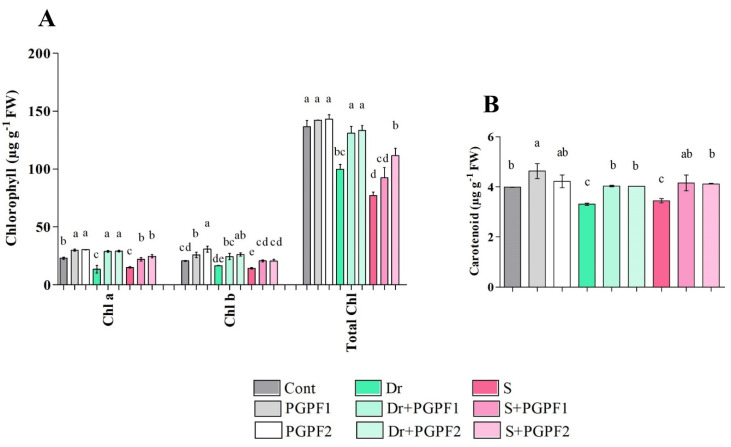
Effects of PGPF inoculation on tomato plant photosynthetic parameters “chlorophyll a; Chla, chlorophyll b; Chlb, total chlorophyll; total Chl, and carotenoid contents” under normal and stress conditions after 12 days of treatment (**A**,**B**). Treatments: Cont (control), PGPF1 (*Actinomucor elegans*), PGPF2 (*Podospora bulbillosa*), Dr (25% Polyethylene glycol), Dr (25% Polyethylene glycol) + PGPF1 (*Actinomucor elegans*), Dr (25% Polyethylene glycol) + PGPF2 (*Podospora bulbillosa*), S (1.5% sodium chloride), S (1.5% sodium chloride) + PGPF1 (*Actinomucor elegans*), and S (1.5% sodium chloride) + PGPF2 (*Podospora bulbillosa*). Values are shown as the means ± SD (*n* = 5) and significant differences at *p* < 0.05 (Tukey test) are indicated by different lowercase letters above the columns.

A marked influence of fungal inoculation as well as drought and salinity stresses on photosynthetic pigments of tomato plants was observed (Figure 4A,B). A significant enhancement in photosynthetic pigments (Chla, Chlb, and carotenoid) was detected in stressed plants subjected to PGPF inoculations in comparison to the un-inoculated stressed plants (Figure 4A). Similarly, PGPF-inoculated stressed plants demonstrated enhanced total chlorophyll contents (Total Chl) in comparison to plants under stress conditions (Figure 4A). A significant reduction in total chlorophyll contents was observed in un-inoculated plants under both drought (26.90%) and salinity (43.59%) conditions. In contrast, PGPF-inoculated plants exhibited an increase in total chlorophyll contents under drought (PGPF1, 23.76%; PGPF2, 25.18%), and salinity (PGPF1, 16.49%; PGPF2, 30.92%) stresses in comparison to the un-inoculated stressed plants (Figure 4A).

#### 3.3.3. Salicylic Acid (SA) Metabolism in Response to PGPF Inoculations and Abiotic Stresses

**Figure 5:**
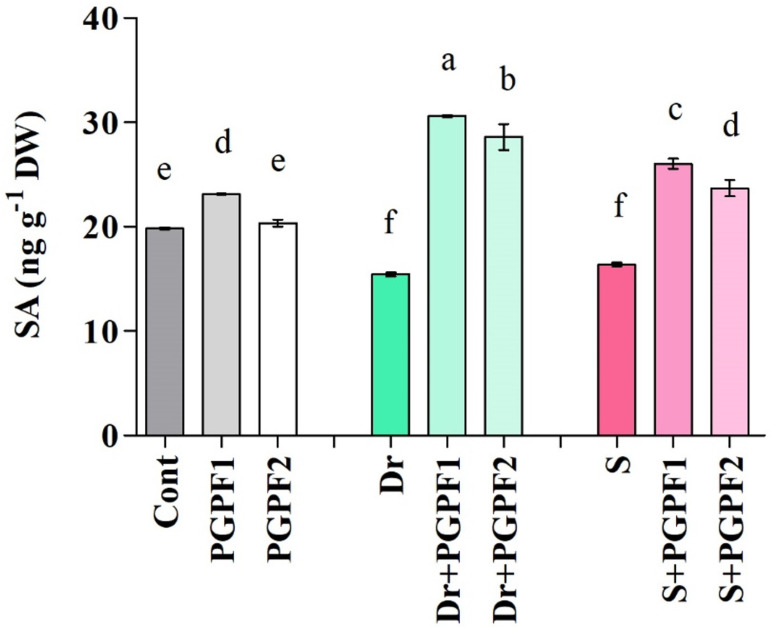
SA content in leaves of tomato plants grown under normal and stress conditions and inoculated with PGPF for 12 days. Treatments: Cont (control), PGPF1 (*Actinomucor elegans*), PGPF2 (*Podospora bulbillosa*), Dr (25% Polyethylene glycol), Dr (25% Polyethylene glycol) + PGPF1 (*Actinomucor elegans*), Dr (25% Polyethylene glycol) + PGPF2 (*Podospora bulbillosa*), S (1.5% sodium chloride), S (1.5% sodium chloride) + PGPF1 (*Actinomucor elegans*), and S (1.5% sodium chloride) + PGPF2 (*Podospora bulbillosa*). Values are shown as the means ± SD (*n* = 5) and significant differences at *p* < 0.05 (Tukey test) are indicated by different lowercase letters above the columns.

Analysis of salicylic acid (SA) levels showed considerable SA reductions under drought and Salinity stresses (Figure 5). Application of PGPF1 and PGPF2 on stressed plants increased SA levels in comparison to the un-inoculated stressed plants. Among the stressed plants, PGPF-inoculated plants showed noticeably higher levels of SA, that is, drought (PGPF1, 49.57%; PGPF2, 46.05%), and salinity stresses (PGPF1, 37.13%; PGPF2, 30.94%) compared to the un-inoculated stressed plants (Figure 5).

#### 3.3.4. Effect of PGPF Inoculations and Abiotic Stresses on Amino Acid Content

**Figure 6:**
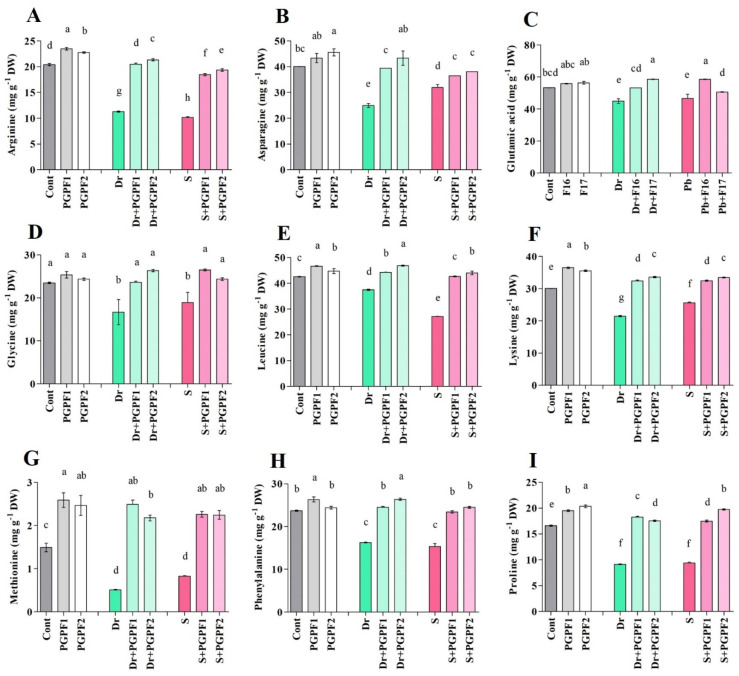
Changes in amino acid contents ((**A**) Arginine, (**B**) Asparagine, (**C**) Glutamic acid, (**D**) Glycine, (**E**) Leucine, (**F**) Lysine, (**G**) Methionine, (**H**) Phenylalanine, (**I**) Proline) in leaves of tomato plants grown under normal and stress conditions and inoculated with PGPF for 12 days. Treatments: Cont (control), PGPF1 (*Actinomucor elegans*), PGPF2 (*Podospora bulbillosa*), Dr (25% Polyethylene glycol), Dr (25% Polyethylene glycol) + PGPF1 (*Actinomucor elegans*), Dr (25% Polyethylene glycol) + PGPF2 (*Podospora bulbillosa*), S (1.5% sodium chloride), S (1.5% sodium chloride) + PGPF1 (*Actinomucor elegans*), and S (1.5% sodium chloride) + PGPF2 (*Podospora bulbillosa*). Values are shown as the means ± SD (*n* = 5) and significant differences at *p* < 0.05 (Tukey test) are indicated by different lowercase letters above the columns.

Drought and salinity stresses caused obvious decreases in amino acid content in tomato plants over 12 days (Figure 6A–I). They also reduced the proline contents by 44.93%, and 43.57%, respectively, in un-inoculated control plants. However, drought and salt stresses could not negatively affect the proline contents in PGPF-inoculated plants. Moreover, 12 days after the application of PGPF1 and PGPF2 on stressed plants, amino acid contents (arginine, asparagine, glutamic acid, glycine, leucine, lysine, methionine, phenylalanine, and proline) were increased compared to un-inoculated stressed plants (Figure 6A–I). Under stress conditions, proline contents increased in PGPF-inoculated plants under drought (PGPF1, 49.94%; PGPF2, 47.87%) and salinity (PGPF1, 46.41%; PGPF2, 52.56%) stresses compared to un-inoculated stressed plants (Figure 6I). 

#### 3.3.5. Hydrogen Peroxide and Malondialdehyde Contents in Tomato Plants Subjected to Varied Treatments

**Figure 7:**
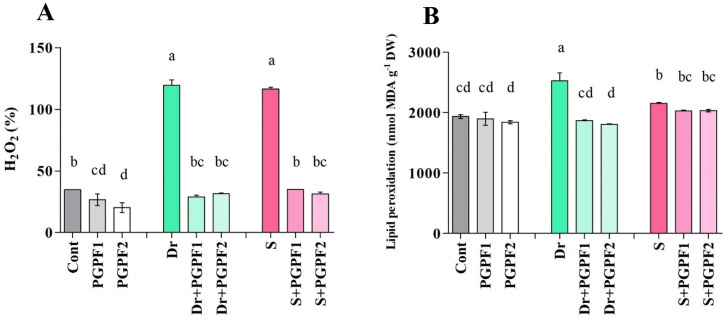
(**A**) H_2_O_2_, and (**B**) Lipid peroxidation contents in leaves of tomato plants grown under normal and stress conditions and inoculated with PGPF for 12 days. Treatments: Cont (control), PGPF1 (*Actinomucor elegans*), PGPF2 (*Podospora bulbillosa*), Dr (25% Polyethylene glycol), Dr (25% Polyethylene glycol) + PGPF1 (*Actinomucor elegans*), Dr (25% Polyethylene glycol) + PGPF2 (*Podospora bulbillosa*), S (1.5% sodium chloride), S (1.5% sodium chloride) + PGPF1 (*Actinomucor elegans*), and S (1.5% sodium chloride) + PGPF2 (*Podospora bulbillosa*). Values are shown as the means ± SD (*n* = 5) and significant differences at *p* < 0.05 (Tukey test) are indicated by different lowercase letters above the columns.

Marked increases were recorded in the hydrogen peroxide (H_2_O_2_) content when plants were exposed to drought and salinity stresses (Figure 7A). H_2_O_2_ contents were enhanced by 70.93% and 70.16% under drought and salt stresses, respectively, as compared with non-stressed plants. There was a significant change in the H_2_O_2_ content between un-inoculated as well as PGPF inoculated plants under stress conditions (Figure 7A). Considerable reductions in H_2_O_2_ contents were recorded in PGPF applied plants under drought (PGPF1, 75.85%; PGPF2, 73.63%), and salinity (PGPF1, 70.02%; PGPF2, 73.12%) stresses (*p* < 0.05).

Application of drought and salinity stresses increased the malondialdehyde (MDA) levels irrespective of the PGPF treatments (Figure 7B). MDA levels raised by 23.40% and 10.08% under drought and salinity stress conditions, respectively. However, PGPF-inoculated plants recorded lower MDA levels under drought (PGPF1, 26.12%; PGPF2, 28.51%), and salinity (PGPF1, 5.59%; PGPF2, 5.44%) in comparison to un-inoculated plants under stress conditions (*p* < 0.05). 

#### 3.3.6. Changes in Protein and Sugar Contents

**Figure 8:**
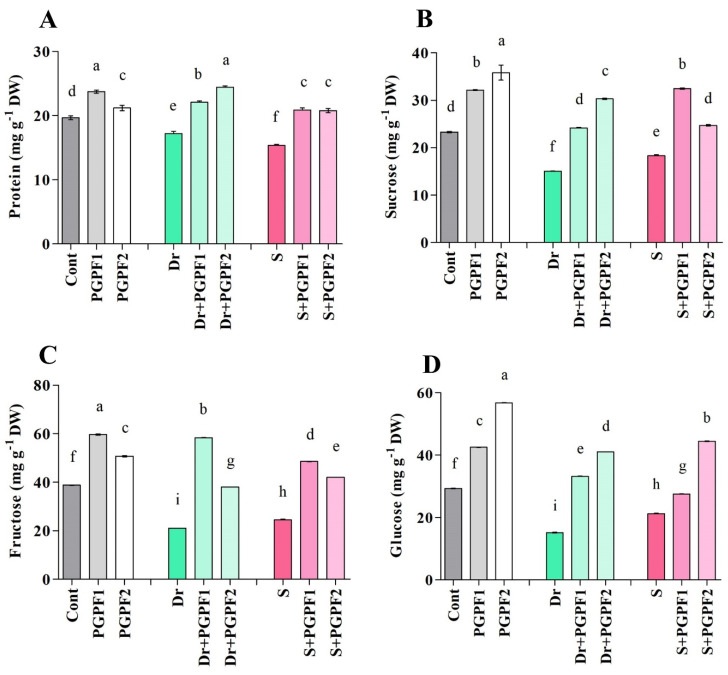
(**A**) Protein, (**B**) Sucrose, (**C**) Fructose, and (**D**) Glucose contents in leaves of tomato plants grown under normal and stress conditions and inoculated with PGPF for 12 days. Treatments: Cont (control), PGPF1 (*Actinomucor elegans*), PGPF2 (*Podospora bulbillosa*), Dr (25% Polyethylene glycol), Dr (25% Polyethylene glycol) + PGPF1 (*Actinomucor elegans*), Dr (25% Polyethylene glycol) + PGPF2 (*Podospora bulbillosa*), S (1.5% sodium chloride), S (1.5% sodium chloride) + PGPF1 (*Actinomucor elegans*), and S (1.5% sodium chloride) + PGPF2 (*Podospora bulbillosa*). Values are shown as the means ± SD (*n* = 5) and significant differences at *p* < 0.05 (Tukey test) are indicated by different lowercase letters above the columns.

Protein contents in tomato plants escalated upon PGPF (PGPF1, 17.18%; PGPF2, 7.22%) applications as compared with un-inoculated plants. However, drought and salt-stressed plants recorded a 12.47%, and 21.84% decrease in protein contents, respectively, compared with the unstressed plants (*p* < 0.05) (Figure 8A). On the other hand, PGPF-inoculated stressed plants recorded improvements in protein contents under drought (PGPF1, 22.08%; PGPF2, 29.62%) and salinity (PGPF1, 26.40%; PGPF2, 26.03%) stresses as compared to the un-inoculated stressed plants. 

Under stress conditions, significant reductions in sucrose, fructose, and glucose levels were observed in tomato plants (Figure 8B–D). For instance, drought and salt-stressed plants recorded lower sucrose levels (drought, 35.47%; salt, 21.37%) in comparison to non-stressed control plants (Figure 8B). However, sucrose, glucose, and fructose levels were highest in PGPF applied plants in both non-stress and stress conditions. For example, PGPF-inoculated stressed plants showed enhanced sucrose levels under drought (PGPF1, 37.81%; PGPF2, 50.39%) and salinity (PGPF1, 43.55%; PGPF2, 25.90%) stresses, compared to that of un-inoculated stressed plants (Figure 8B).

#### 3.3.7. The Activity of Non-Enzymatic and Enzymatic Antioxidants 

**Figure 9:**
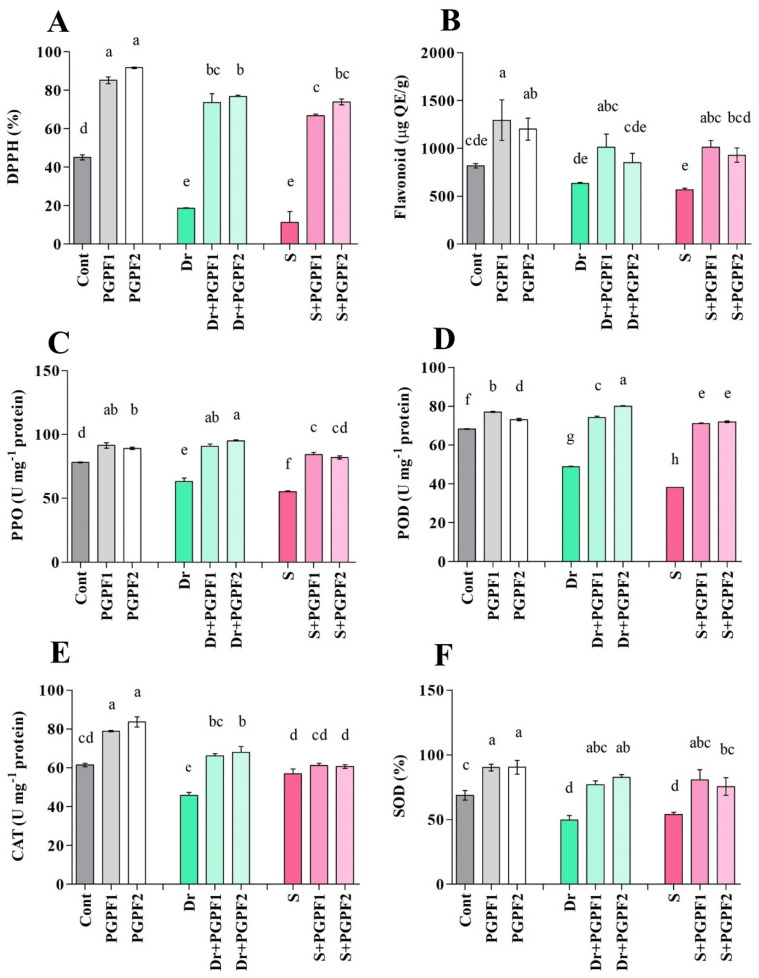
Antioxidant contents ((**A**) DPPH (2,2-diphenyl-1-picrylhydrazyl), (**B**) flavonoid, (**C**) PPO (Polyphenol oxidase), (**D**) POD (Peroxidase), (**E**) CAT (Catalase) and (**F**) SOD (Superoxide dismutase)) in leaves of tomato plants grown under normal and stress conditions and inoculated with PGPF for 12 days. Treatments: Cont (control), PGPF1 (*Actinomucor elegans*), PGPF2 (*Podospora bulbillosa*), Dr (25% Polyethylene glycol), Dr (25% Polyethylene glycol) + PGPF1 (*Actinomucor elegans*), Dr (25% Polyethylene glycol) + PGPF2 (*Podospora bulbillosa*), S (1.5% sodium chloride), S (1.5% sodium chloride) + PGPF1 (*Actinomucor elegans*), and S (1.5% sodium chloride) + PGPF2 (*Podospora bulbillosa*). Values are shown as the means ± SD (*n* = 5) and significant differences at *p* < 0.05 (Tukey test) are indicated by different lowercase letters above the columns.

In the current study, we evaluated the status of non-enzymatic antioxidants including DPPH (2,2-diphenyl-1-picrylhydrazyl), flavonoid, PPO (Polyphenol oxidase), and enzymatic antioxidants such as POD (Peroxidase), CAT (Catalase), and SOD (Superoxide dismutase) in the PGPF inoculated and un-inoculated plants under non-stress and stress conditions (Figure 9A–F). Tomato seedlings showed decreased DPPH activity under stress conditions, while seedlings inoculated with fungal strains demonstrated enhanced DPPH activity under stress conditions. For instance, DPPH activity significantly increased in PGPF1-inoculated plants by 74.84%, and 83.20% under drought and salinity stresses, respectively, as compared to the DPPH activity detected in un-inoculated stressed plants (Figure 9A). 

In the case of flavonoids, a considerable reduction of flavonoid activity was evident in stressed plants. However, treatment with the fungal strains increased flavonoid activity in tomato plants under stress conditions (*p* < 0.05) (Figure 9B). Furthermore, the same trend was observed with PPO and POD activities, which reduced clearly under drought and salinity stresses. Contrary to this, the stressed plants showed a substantial enhancement of the PPO and POD activities following treatments with fungal strains (PGPF1 and PGPF2). For example, PGPF2 treatment resulted in increased POD activity in drought (38.99%) and salinity (46.95%) in the stressed plants (*p* < 0.05) (Figure 9D).

Drought and salinity stresses caused a significant reduction in CAT and SOD activity in tomato plants. However, the fungal strains were found to cause significant increase in CAT and SOD activity in inoculated plants compared to un-inoculated stressed plants (Figure 9E,F).

#### 3.3.8. PGPF Modifies Nutrient and Sodium Contents in Tomato Plants 

**Figure 10:**
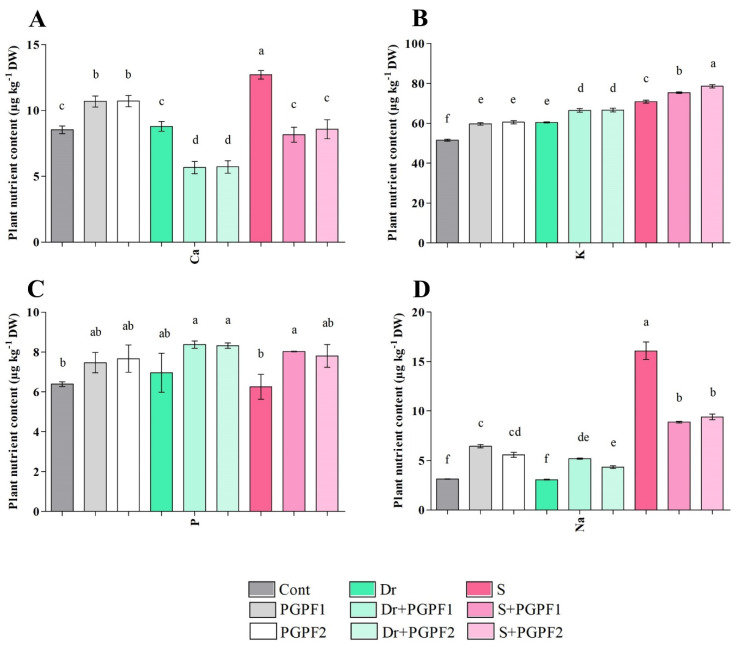
Nutrient contents ((**A**) Ca (Calcium), (**B**) K (Potassium), (**C**) P (Phosphorous) and (**D**) Na (Sodium)) in leaves of tomato plants grown under normal and stress conditions and inoculated with PGPF for 12 days. Treatments: Cont (control), PGPF1 (*Actinomucor elegans*), PGPF2 (*Podospora bulbillosa*), Dr (25% Polyethylene glycol), Dr (25% Polyethylene glycol) + PGPF1 (*Actinomucor elegans*), Dr (25% Polyethylene glycol) + PGPF2 (*Podospora bulbillosa*), S (1.5% sodium chloride), S (1.5% sodium chloride) + PGPF1 (*Actinomucor elegans*), and S (1.5% sodium chloride) + PGPF2 (*Podospora bulbillosa*). Values are shown as the means ± SD (*n* = 5) and significant differences at *p* < 0.05 (Tukey test) are indicated by different lowercase letters above the columns.

**Figure 11:**
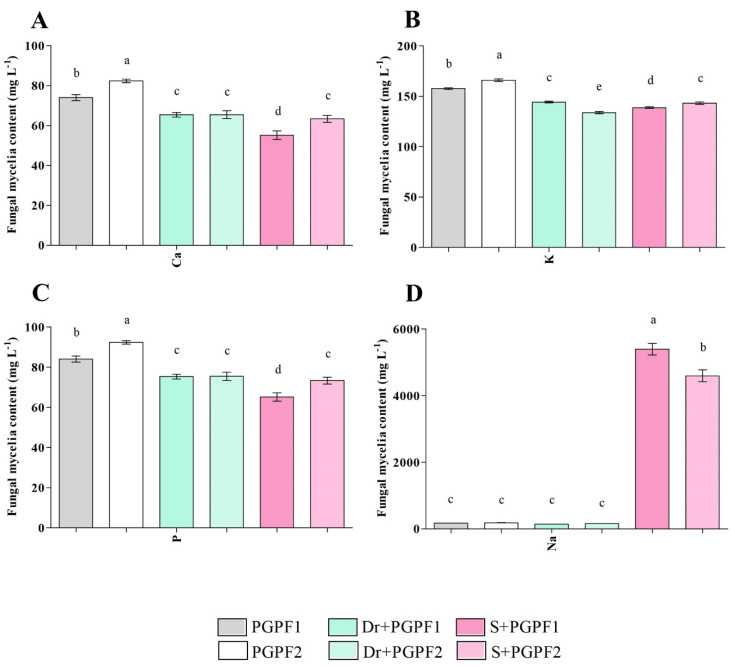
Nutrient contents ((**A**) Ca (Calcium), (**B**) K (Potassium), (**C**) P (Phosphorous) and (**D**) Na (Sodium)) in fungal mycelia under varied treatments during 12 days. Treatments: PGPF1 (*Actinomucor elegans*), PGPF2 (*Podospora bulbillosa*), Dr (25% Polyethylene glycol) + PGPF1 (*Actinomucor elegans*), Dr (25% Polyethylene glycol) + PGPF2 (*Podospora bulbillosa*), S (1.5% sodium chloride) + PGPF1 (*Actinomucor elegans*), and S (1.5% sodium chloride) + PGPF2 (*Podospora bulbillosa*). Values are shown as the means ± SD (*n* = 5) and significant differences at *p* < 0.05 (Tukey test) are indicated by different lowercase letters above the columns.

The concentration of Ca, K, P, and Na was determined in tomato plants in the absence and presence of the fungal strains (PGPF1 and PGPF2) under non-stress and stress conditions (Figure 10A–D). Increased Ca, K, and P contents were observed in the unstressed plants inoculated with fungal strains compared to the relevant contents in control plants. Moreover, PGPF-inoculated plants showed improved K and P contents and reduced Ca content under stress conditions. Salt-stressed plants had accumulated higher contents of Na compared with unstressed control plants. Interestingly, PGPF-inoculated plants demonstrated lower Na accumulation compared to the plants under salt stress conditions (Figure 10D). Na uptake by fungal mycelia is presented in Figure 11D; these results validate the recovery role of PGPF1 and PGPF2. The removal efficiency of Na by fungal strains was 96.79% (PGPF1), and 96.0% (PGPF2). 

### 3.4. Expression Pattern of Drought and Salinity Responsive Genes in Tomato Plants in Response to Application of PGPFs and Abiotic Stresses

**Figure 12:**
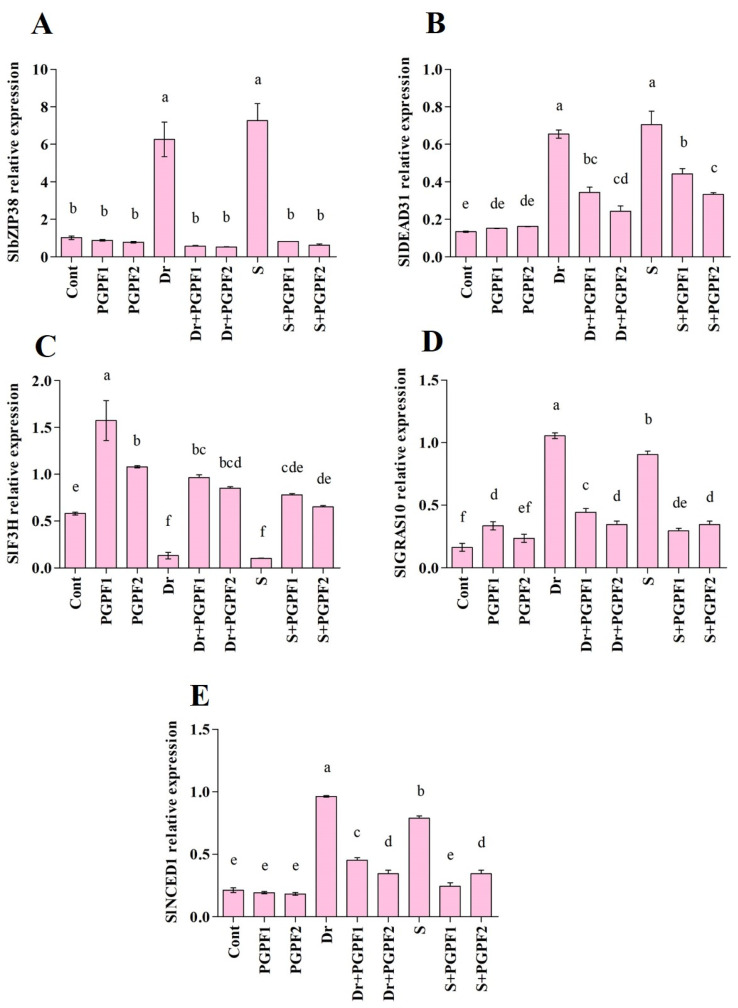
Real-time expression analysis of (**A**) *SlbZIP38*, (**B**) *SlDEAD31*, (**C**) *SlF3H*, (**D**) *SlGRAS10*, and (**E**) *SlNCED1* in leaves of tomato plants grown under normal and stress conditions and inoculated with PGPF for 12 days. Treatments: Cont (control), PGPF1 (*Actinomucor elegans*), PGPF2 (*Podospora bulbillosa*), Dr (25% Polyethylene glycol), Dr (25% Polyethylene glycol) + PGPF1 (*Actinomucor elegans*), Dr (25% Polyethylene glycol) + PGPF2 (*Podospora bulbillosa*), S (1.5% sodium chloride), S (1.5% sodium chloride) + PGPF1 (*Actinomucor elegans*), and S (1.5% sodium chloride) + PGPF2 (*Podospora bulbillosa*). Values are shown as the means ± SD (*n* = 5) and significant differences at *p* < 0.05 (Tukey test) are indicated by different lowercase letters above the columns.

In the current experiment, *SlbZIP38* transcription was induced and highly expressed in tomato plants after drought and salt stresses as compared with unstressed plants. The results indicate that the expression of *SlbZIP38* was upregulated by both abiotic stresses. When treated with PGPFs, *SlbZIP38* transcripts were downregulated under drought and salinity. The PGPFs implementation reduced *SlbZIP38* expression under drought (PGPF1, 90.87%; PGPF2, 91.67%) and salt (PGPF1, 88.68%; PGPF2, 91.44%) stresses (Figure 12A).

Quantitative RT-PCR analysis demonstrated that *SlDEAD31* expression was promoted apparently in stressed tomato plants. In addition, the expression of *SlDEAD31* was affected by PGPF treatments and downregulated under both stresses (Figure 12B). For instance, PGPF1 implementation reduced *SlDEAD31* transcripts by about 47.63%, and 37.16% under drought and salinity stresses, respectively, in comparison to stressed plants alone.

To evaluate the effect of PGPF application on *SlF3H*, we examined the level of *SlF3H* in tomato plants subjected to abiotic stresses and normal conditions (Figure 12C). During unstressed conditions, variations were shown in the *SlF3H* gene expression in control and PGPF-treated plants. The change was more pronounced in stressed plants. The *SlF3H* expression dramatically declined in tomato plants following drought and salinity treatments. Plants treated with PGPF displayed a rise in *SlF3H* expression as compared to untreated stressed plants under drought (PGPF1, 86.60%; PGPF2, 84.80%), and salinity (PGPF1, 87.29%; PGPF2, 84.74%).

As shown in Figure 12D, *SlGRAS10* expression was relatively higher in drought and salinity-stressed plants, by 84.64%, and 82.09% respectively, in contrast with the expression discerned in the control plants. On the other hand, weak expression of *SlGRAS10* was recorded in PGPF-treated plants affected by abiotic stresses. *SlGRAS10* was expressed at a lower level in PGPF-treated plants under drought (PGPF1, 58.0%; PGPF2, 67.48%), and salinity (PGPF1, 67.62%; PGPF2, 62.09%) (Figure 12D). 

To confirm whether the expression of *SlNCED1* was affected in tomato plants, we thus analyzed its transcription pattern under normal and stress conditions. The results showed that *SlNCED1* expression level fluctuated under varied treatments (Figure 12E). The expression of *SlNCED1* increased sharply and was highest in drought (77.98%), and salt (73.09%) stressed plants. Conversely, *SlNCED1* expression declined to a low level after the application of PGPF1 and PGPF2. The *SlNCED1* expression was downregulated in PGPF-treated plants under both drought (PGPF1, 52.95%; PGPF2, 64.38%), and salt PGPF1, 69.16%; PGPF2, 56.47%) stresses.

## 4. Discussion

Research in the last couple of years has shown that plant growth-promoting fungi (PGPF) play an indispensable function in the modulation of plant acclimatization to environmental stresses [57]. This study focused on investigating the effects of *Actinomucor elegans* and *Podospora bulbillosa* on tomato seedlings under stress and non-stress conditions. PGPF-inoculated seedlings were found to be recovered, and both PGPFs enhanced drought and salt endurance as noticed from the improved growth traits. Plant growth assessment experiments using fungal strains demonstrated that the application of both fungi had enhanced plant biometric parameters (height, lateral root branching, stem diameter, leaf area, plant fresh/dry weight, and root fresh/dry weight) of unstressed/stressed seedlings compared to stressed plants in the absence of the fungal strains. In addition, chlorophyll and carotenoid contents were raised in the PGPF-treated plants. It should be noted that our in vitro studies confirmed IAA and ammonia production by both fungal strains. It has been confirmed that ammonia, as a main inorganic nitrogen source, improves plant growth, influences genes involved in metabolic processes and scavenging of reactive oxygen species (ROS), and triggers phosphorylation events that affect enzymes involved in amino acid and protein synthesis [58]. Moreover, ammonia changes root system architecture by hindering root elongation, inducing lateral root branching and root hair development [59]. Sun, et al. [60] indicated that fungal-produced IAA can stimulate lateral root formation and root hair development which led to increased nutrient absorption by the associated plant. PGPF-inoculated plants exhibited enhanced K and P contents and alleviated Ca, and Na contents relative to the effects detected in un-inoculated stressed plants. Calcium (Ca), recognized as an essential second manager in plants, controls plant growth and responses to abiotic stresses, pathogens, and mechanical wounds [61]. Moreover, calcium signals are involved in numerous responses to plant hormones such as abscisic acid, jasmonic acid, and gibberellic acid [62]. Overall, these improvements in non-stressed and stressed plants could be due to fungal metabolite production, elevated absorption of nutrients from the rhizosphere, and the impact of obtained nutrients on varied biosynthetic pathways.

DEAD-box proteins are conserved RNA helicases that are associated with RNA metabolism and have roles in varied cellular mechanisms such as plant growth, development, and response to abiotic stresses [63,64]. It has been demonstrated that these proteins take part in plant responses to abiotic stresses such as salt, drought, temperature, and cold [63,65]. We explored the expression pattern of *SlDEAD31* during tomato development in response to drought and salinity stresses. Zhu, et al. [55] reported high expression of *SlDEAD31* in tomato plants exposed to salinity and drought stresses, which was consistent with our findings. On the other hand, PGPF-inoculated plants showed reduced expression of *SlDEAD31* under stress conditions. This could be due to the stress soothing effect of PGPF.

Osmotic stress elicitation of ABA aggregation is a distinguished fact [13]. Abscisic acid has extensive roles in plant growth, one of which is managing plant water balance and response to stresses [66]. The expression of varied plant genes has been demonstrated to be modulated by water deficit and salt stress. A considerable group of these gens is responsive to ABA regulation as well [13]. Zeaxanthin epoxidase (*ZEP*), 9-*cis*-epoxycarotenoid dioxygenase (*NCED*), and abscisic aldehyde oxidase (*AAO*) are the most ABA biosynthesis genes [67,68]. Previous studies have shown that the endogenous concentration of ABA in plants is enhanced via two distinct pathways [69]. 9-*cis*-epoxycarotenoid dioxygenase (*NCED*) has been recognized as the primary pathway and key regulatory enzyme in ABA biosynthetic pathway [68,70,71]. Evidence indicates that enhanced *NCED* expression level improves ABA biosynthesis, raises ABA accumulation under abiotic stresses, and increases stress endurance in plants [72,73,74]. In this study, *SlNCED1* was rapidly induced by drought and salinity stresses, which led to an elevated accumulation of ABA in stressed plants. On the other hand, PGPF-inoculated plants showed reduced expression of *SlNCED1* and subsequently decreased ABA levels under stress conditions. Numerous studies have reported reduced ABA levels in stressed plants associated with fungi [75,76,77,78]. It is reasonable to hypothesize that PGPF (*A. elegans* and *P. bulbillosa*) repressed ABA-dependent transcription factors and stimulated ABA-independent pathways to mediate stress responses and combat abiotic stresses. 

Salicylic acid, an intrinsically occurring phenolic compound, plays a role in several plant physiological and metabolic responses, such as photosynthesis, flowering, root initiation and growth, protection responses, seed germination, proline metabolism, and respiration [72,79,80]. It has been shown that salicylic acid is primarily a phytohormone associated with responses to abiotic stress and promotes plant tolerance to abiotic stresses such as ozone, heat, salt, cold, drought, UV radiation, and metal [80,81,82,83,84,85]. Salicylic acid works with the other plant hormones by triggering antioxidants and enzymes to suppress varied abiotic stresses and increase plant tolerance to them [80]. In this study, we observed enhanced salicylic acid production in PGPF-inoculated plants under non-stress and stress conditions. Miura and Tada [86] reported that the cell redox reactions are modulated by salicylic acid with reduced augmentation of reactive oxygen species (ROS). Reactive oxygen species (ROS) mainly act as signal transduction molecules that manage varied pathways throughout plant adaptation to stress [87]. Water deficit and salinity result in the augmentation of considerable amounts of reactive oxygen species (ROS) generation [88]. At excessive production, ROS are toxic and can induce oxidative damage, modify DNA, cause membrane peroxidation and protein degradation, obstruct metabolic functions, actuate program cell death, and hinder enzymes [89]. Indeed, we observed a higher accumulation of H_2_O_2_ and MDA in stressed plants, which can be attributed to the unbalance rate of ROS generation and elimination [90,91]. Moreover, analyses revealed that PGPF inoculation influences the content of H_2_O_2_ and MDA and significantly reduced their accumulation in stressed plants at later stages, which agrees with previous studies [92,93,94,95]. This ROS reduction could be due to elevated salicylic acid production in PGPF-inoculated stressed plants to modulate ROS accumulation, leading to decreased ROS content. 

The basic leucine zipper (bZIP) family is considered one of the biggest transcription factor families in plants. Various studies have demonstrated that transcription factor (bZIP) has an important role in plant responses to abiotic stresses [39,96,97]. It has been confirmed that bZIP genes act in ABA-regulated responses to varied stresses such as drought and salinity [96]. In this study, the expression of *SlbZIP38* was strongly induced by drought and salinity stresses and led to its accumulation. Pan, et al. [39] indicated that enhanced upregulation of *SlbZIP38* led to decreased drought and salinity endurance in tomato plants. Moreover, they observed reduced chlorophyll and proline contents but increased malondialdehyde content in stressed plants, which goes along with our results. On the other hand, PGPF-inoculated plants exhibited repressed *SlbZIP38* under stress conditions. It is reasonable to assume that PGPF downregulated *SlbZIP38* expression and subsequently decreased ABA content, increased chlorophyll and proline contents and reduced malondialdehyde content in plants subjected to drought and salinity stresses. 

Plants are commonly confronted with an extensive range of harsh environmental conditions such as salinity, drought, or heat. These stresses happen not one by one but in combination and they may co-occur with assaults by varied pathogens [98]. Therefore, plants have established sophisticated ways to evade or persist in the disastrous consequences. The GRAS protein family (name derived from GAI, RGA, SCR) usually functions as a transcription factor and plays a significant role under abiotic and biotic stresses [99]. The GRAS family genes have a role in plant growth, gibberellins signal transduction, shoot meristem maintenance, and root radial organization [100,101,102,103]. Moreover, they manage plant retorts to abiotic and biotic stresses [104,105,106]. We observed enhanced expression of *GRAS* under drought and salinity stresses, whereas PGPF application reduced *GRAS* accumulation in stressed plants. Habib, et al. [56] showed that reduced expression of *GRAS* enhances tomato plant endurance to salt and drought stresses by improved chlorophyll content, flavonoid biosynthesis, and ROS scavenging enzymes. In this study, PGPF (*A. elegans* and *P. bulbillosa*) reduced *GRAS* expression in stressed plants which led to enhanced chlorophyll and antioxidant activities. Consequently, theses physiological changes promote the plant’s ability to endure under environmental stresses. 

Stress occurrence, duration, and intensity are completely unpredictable and therefore plants have devised procedures to sustain their growth, metabolism, and productivity in response to environmental stresses [107]. To detoxify excess ROS and restore the redox balance, plants trigger endogenous procedures that engage enzymatic and non-enzymatic defense mechanisms [108,109]. In this study, the antioxidants’ function declined in stressed plants but improved following the use of PGPF in stressed plants. It has been found that plant colonization by fungal strains increases the efficiency of the host antioxidative system by controlling the production or activity of varied antioxidants [110]. Ancreased level of antioxidants is positively conformed to greater endurance and protection levels against abiotic stresses. Caverzan, et al. [111] indicated that enhancement in antioxidant activity and reduction in oxidative damage are closely connected. This rise in antioxidant activity implies the important role of PGPFs in regulating plant responses to water deficit and salinity by scavenging excessive ROS, alleviating oxidative harm, and ameliorating endurance to oxidative stress. 

Flavanone 3-hydroxylase (*F3H*) is a key enzyme that catalyzes a crucial stage in flavonoid biosynthesis [112]. Previous studies have demonstrated that *F3H* not only manages flavonoid quantities but also takes part in endurance to stresses [52,113]. To evaluate the response of *F3H* to selected abiotic stresses, we evaluated the effects of drought and salinity on its transcription pattern in tomato plants. We detected noticeable reduction in *F3H* expression levels in salinity and drought-stressed plants. However, PGPF-inoculated plants exhibited enhanced expression of *F3H*. Our biochemical and molecular analyses showed that PFPF-inoculated plants displayed enhanced flavonoid content at the end of the experiment. Meng, et al. [53] reported that *F3H* upregulation led to higher flavonoid content, better growth/survival, and lower H_2_O_2_/MDA accumulation in stressed plants. Due to performing as a ROS scavenging antioxidant, flavonoid assists plants to withstand varied abiotic stress conditions [111,114]. These findings suggest that PGPF upregulated *F3H* expression and subsequently *F3H* induces flavonoid biosynthesis and increases tolerance to drought and salinity stresses. 

Sugars are crucial biomolecules working as the main carbon reservoir in plants [115]. They participate in varied physiological procedures such as growth, development, metabolism, and reproduction [116]. They also function as osmolytes and sustain the osmotic capability and turgor of plant cells during abiotic stresses [115]. Adverse environmental conditions can dwindle leaf sugar content and as a consequence provoke physiochemical modifications [117]. In the present study, the soluble sugar content (glucose, fructose, and sucrose) was high in tomato plants in presence of PGPF. A marked increase in glucose, fructose, and sucrose was discerned in tomato seedlings inoculated with PGPF under non-stress and stress conditions. Vergara, et al. [118] reported higher sugar content in rice plants inoculated with dark septate fungus from order *Pleosporales*. It has been confirmed that the high escalation of sugar in plants demonstrates an immensely protective mechanism against oxidative damage caused by unfavorable conditions [119,120]. Therefore, this indicates that *A. elegans* and *P. bulbillosa* had a noticeable influence on the sugar accumulation in stressed seedlings, which act as an osmolyte to govern osmotic adjustments, scavenge ROS and generate tolerance in plants.

Amino acids participate in the metabolism of varied secondary products in plants to eliminate stress-caused damage in plants and bestow endurance in them [121]. The findings from the current study showed reduced amino acid content in tomato under abiotic stresses. The application of PGPF improved amino acid values in stressed seedlings within the rehabilitation time. This amino acid augmentation may take part in several processes [48,122]. It has been demonstrated that proline takes part as osmoprotecting molecules in higher plants [123]. An increased level of proline was observed in PGPF-treated seedlings in response to water stress and salinity which was in agreement with previous studies [114,124]. This enhanced proline content could be the *A. elegans* and *P. bulbillosa* strategy to confer protection from these stresses and reduce unfavorable effects of free radical activity in stressed plants. It has been confirmed that the awakening of proline biosynthesis ameliorates protein turnover, scavenges reactive oxygen species, and helps plants to withstand abiotic stress [125,126,127]. Bettered proline content has been tightly engaged in stabilized membrane and protein structure, minimized cell impairment, and restored plant growth under environmental stress [128,129]. Our results suggest that PGPF application generated enhanced amino acids, and eminently proline content to protect plants from stress conditions. 

## 5. Conclusions

The utilization of *A. elegans* and *P. bulbillosa* not only exacerbated tomato liveliness under water deficit and salinity but also unquestionably actuated tomato tolerance to these abiotic stresses. The potential of *A. elegans* and *P. bulbillosa* under abiotic generated stress governed host growth by means of relieving water deficit and salinity escalation in tomato plants. Likewise, *A. elegans* and *P. bulbillosa* execution transformed host biochemistry to eliminate the disastrous consequences of abiotic stresses. In this report, water deficit and salt stresses restrained some genes, albeit *A. elegans* and *P. bulbillosa* was apt for confronting the repression consequence of drought and salt stresses and revived of varied repressed genes. *A. elegans* and *P. bulbillosa* affected the expression of stress-related genes explicitly *SlbZIP38*, *SlDEAD31*, *SlF3H*, *SlGRAS10*, and *SlNCED1*. Generally, the information provided in this study demonstrated that *A. elegans* and *P. bulbillosa* take part in the modulation of adaptive developmental responses to detrimental stresses. This makes *A. elegans* and *P. bulbillosa* suitable candidates which potentially can be used to improve the agronomic performance and adaptation of tomato and other important crops. 

## Figures and Tables

**Table 1:** Experimental work plan.

Symbol	Treatment
Cont	irrigated with sterile distilled water
PGPF1	irrigated with *Actinomucor elegans*
PGPF2	irrigated with *Podospora bulbillosa*
Dr	irrigated with 25%PEG (−0.73 Mpa)
Dr + PGPF1	irrigated with 25%PEG (−0.73 Mpa) + *Actinomucor elegans*
Dr + PGPF2	irrigated with 25%PEG (−0.73 Mpa) + *Podospora bulbillosa*
S	irrigated with 1.5% NaCl
S + PGPF1	irrigated with 1.5% NaCl + *Actinomucor elegans*
S + PGPF2	irrigated with 1.5% NaCl + *Podospora bulbillosa*

## Data Availability

Not applicable.

## Supplementary Materials

The following supporting information can be downloaded at: https://www.mdpi.com/article/10.3390/jof8080785/s1, Figure S1. (A) Siderophore production, (B) Ammonia production, (C) IAA production; Figure S2. Drought (A,B) and Salt (C,D) tolerance ability of the selected fungal strains associated with *Solanum lycopersicum* in this study; Figure S3. pH tolerance ability of the selected fungal strains associated with *Solanum lycopersicum* in this study (A,B); Figure S4. Effects of PGPF inoculation on tomato plant growth parameters under normal and stress conditions after 12 days of treatment (A–I); Table S1. Primers used for relative gene expression analysis.

## References

[1] Zhao Y., Ma Q., Jin X., Peng X., Liu J., Deng L., Yan H., Sheng L., Jiang H., Cheng B. (2014). A Novel Maize Homeodomain–Leucine Zipper (HD-Zip) I Gene, Zmhdz10, Positively Regulates Drought and Salt Tolerance in Both Rice and Arabidopsis. Plant Cell Physiol..

[2] Anjum S.A., Xie X.-Y., Wang L.-C., Saleem M.F., Man C., Lei W. (2011). Morphological, physiological and biochemical responses of plants to drought stress. Afr. J. Agric. Res..

[3] Böttner L., Grabe V., Gablenz S., Böhme N., Appenroth K.J., Gershenzon J., Huber M. (2021). Differential localization of flavonoid glucosides in an aquatic plant implicates different functions under abiotic stress. Plant Cell Environ..

[4] Keep T., Sampoux J.-P., Barre P., Blanco-Pastor J.-L., Dehmer K.J., Durand J.-L., Hegarty M., Ledauphin T., Muylle H., Roldán-Ruiz I. (2021). To grow or survive: Which are the strategies of a perennial grass to face severe seasonal stress?. Funct. Ecol..

[5] Wani S.H., Kumar V., Shriram V., Sah S.K. (2016). Phytohormones and their metabolic engineering for abiotic stress tolerance in crop plants. Crop J..

[6] Saddiq M.S., Iqbal S., Hafeez M.B., Ibrahim A.M., Raza A., Fatima E.M., Baloch H., Woodrow P., Ciarmiello L.F. (2021). Effect of Salinity Stress on Physiological Changes in Winter and Spring Wheat. Agronomy.

[7] Abo Nouh F., Abu-Elsaoud A., Abdel-Azeem A.J.M.B. (2021). The role of endophytic fungi in combating abiotic stress on tomato. Microb. Biosyst..

[8] Zahra N., Mahmood S., Raza Z.A. (2018). Salinity stress on various physiological and biochemical attributes of two distinct maize (*Zea mays* L.) genotypes. J. Plant Nutr..

[9] Yadav T., Kumar A., Yadav R., Yadav G., Kumar R., Kushwaha M. (2020). Salicylic acid and thiourea mitigate the salinity and drought stress on physiological traits governing yield in pearl millet-wheat. Saudi J. Biol. Sci..

[10] Allen M., Babiker M., Chen Y., Coninck H.D., Connors S., Diemen R.V., Dube O., Ebi K., Engelbrecht F., Ferrat M. (2018). Global Warming of 1.5 °C—Summary for Policymakers.

[11] Dey R., Lewis S.C., Arblaster J.M., Abram N.J. (2019). A review of past and projected changes in Australia’s rainfall. Wiley Interdiscip. Rev. Clim. Change.

[12] Boretti A., Rosa L. (2019). Reassessing the projections of the World Water Development Report. npj Clean Water.

[13] Zhu J.-K. (2002). Salt and drought stress signal transduction in plants. Annu. Rev. Plant Biol..

[14] Abdul Rahman N.S.N., Abdul Hamid N.W., Nadarajah K. (2021). Effects of Abiotic Stress on Soil Microbiome. Int. J. Mol. Sci..

[15] Jahagirdar S., Kambrekar D., Navi S., Kunta M. (2019). Plant growth-promoting fungi: Diversity and classification. Bioactive Molecules in Plant Defense.

[16] Banhara A., Ding Y., Kühner R., Zuccaro A., Parniske M. (2015). Colonization of root cells and plant growth promotion by Piriformospora indica occurs independently of plant common symbiosis genes. Front. Plant Sci..

[17] Naznin H.A., Kiyohara D., Kimura M., Miyazawa M., Shimizu M., Hyakumachi M. (2014). Systemic resistance induced by volatile organic compounds emitted by plant growth-promoting fungi in Arabidopsis thaliana. PLoS ONE.

[18] Nagaraju A., Sudisha J., Murthy S.M., Ito S.-I. (2012). Seed priming with Trichoderma harzianum isolates enhances plant growth and induces resistance against Plasmopara halstedii, an incitant of sunflower downy mildew disease. Australas. Plant Pathol..

[19] Frąc M., Hannula S.E., Bełka M., Jędryczka M. (2018). Fungal biodiversity and their role in soil health. Front Microbiol..

[20] Hosseyni Moghaddam M.S., Safaie N., Soltani J., Hagh-Doust N. (2021). Desert-adapted fungal endophytes induce salinity and drought stress resistance in model crops. Plant Physiol. Biochem..

[21] Sousaraei N., Mashayekhi K., Mousavizadeh S.J., Akbarpour V., Medina J., Aliniaeifard S. (2021). Screening of tomato landraces for drought tolerance based on growth and chlorophyll fluorescence analyses. Hortic. Environ. Biotechnol..

[22] Collins E.J., Bowyer C., Tsouza A., Chopra M. (2022). Tomatoes: An extensive review of the associated health impacts of tomatoes and factors that can affect their cultivation. Biology.

[23] Dong Z., Men Y., Liu Z., Li J., Ji J. (2020). Application of chlorophyll fluorescence imaging technique in analysis and detection of chilling injury of tomato seedlings. Comput. Electron. Agric..

[24] Kurakov A.V., Lavrent’ev R.B., Nechitailo T.Y., Golyshin P.N., Zvyagintsev D.G. (2008). Diversity of facultatively anaerobic microscopic mycelial fungi in soils. Microbiology.

[25] Wang H.L., Vespa J.B., Hesseltine C.W. (1974). Acid Protease Production by Fungi Used in Soybean Food Fermentation. Appl. Microbiol..

[26] Charria-Girón E., Surup F., Marin-Felix Y. (2022). Diversity of biologically active secondary metabolites in the ascomycete order Sordariales. Mycol. Prog..

[27] Naziya B., Murali M., Amruthesh K.N. (2020). Plant Growth-Promoting Fungi (PGPF) Instigate Plant Growth and Induce Disease Resistance in Capsicum annuum L. upon Infection with Colletotrichum capsici (Syd.) Butler & Bisby. Biomolecules.

[28] Kavamura V.N., Robinson R.J., Hayat R., Clark I.M., Hughes D., Rossmann M., Hirsch P.R., Mendes R., Mauchline T.H. (2019). Land Management and Microbial Seed Load Effect on Rhizosphere and Endosphere Bacterial Community Assembly in Wheat. Front. Microbiol..

[29] Bhattacharyya C., Banerjee S., Acharya U., Mitra A., Mallick I., Haldar A., Haldar S., Ghosh A., Ghosh A. (2020). Evaluation of plant growth promotion properties and induction of antioxidative defense mechanism by tea rhizobacteria of Darjeeling, India. Sci. Rep..

[30] Alexander D., Zuberer D. (1991). Use of chrome azurol S reagents to evaluate siderophore production by rhizosphere bacteria. Biol. Fertil. Soils.

[31] Leo Daniel A., Praveen Kumar G., Desai S., Mir Hassan A. (2011). In vitro characterization of Trichoderma viride for abiotic stress tolerance and field evaluation against root rot disease in *Vigna mungo* L.. J. Biofertil. Biopestici..

[32] Al-Sadi A.M., Al-Masoodi R.S., Al-Ismaili M., Al-Mahmooli I.H. (2015). Population structure and development of resistance to hymexazol among Fusarium solani populations from date palm, citrus and cucumber. J. Phytopathol..

[33] Horisawa S., Sakuma Y., Doi S. (2013). Identification and species-typing of wood rotting fungi using melting curve analysis. J. Wood Sci..

[34] White T.J., Bruns T., Lee S., Taylor J., Innis M., Gelfand D., Sninsky J. (1990). PCR Protocols: A Guide to Methods and Applications.

[35] Maharachchikumbura S.S.N., Wanasinghe D.N., Elgorban A.M., Al-Rejaie S.S., Kazerooni E.A., Cheewangkoon R. (2022). Brunneosporopsis yunnanensis gen. et sp. nov. and Allocryptovalsa xishuangbanica sp. nov., New Terrestrial Sordariomycetes from Southwest China. Life.

[36] Silvestro D., Michalak I. (2012). raxmlGUI: A graphical front-end for RAxML. Org. Divers. Evol..

[37] Zhang S., Gan Y., Xu B. (2015). Biocontrol potential of a native species of Trichoderma longibrachiatum against Meloidogyne incognita. Appl. Soil Ecol..

[38] Bharti N., Pandey S.S., Barnawal D., Patel V.K., Kalra A. (2016). Plant growth promoting rhizobacteria Dietzia natronolimnaea modulates the expression of stress responsive genes providing protection of wheat from salinity stress. Sci. Rep..

[39] Pan Y., Hu X., Li C., Xu X., Su C., Li J., Song H., Zhang X., Pan Y. (2017). SlbZIP38, a tomato bZIP family gene downregulated by abscisic acid, is a negative regulator of drought and salt stress tolerance. Genes.

[40] Jini D., Joseph B. (2017). Physiological Mechanism of Salicylic Acid for Alleviation of Salt Stress in Rice. Rice Sci..

[41] Toderich K.N., Mamadrahimov A.A., Khaitov B.B., Karimov A.A., Soliev A.A., Nanduri K.R., Shuyskaya E.V. (2020). Differential Impact of Salinity Stress on Seeds Minerals, Storage Proteins, Fatty Acids, and Squalene Composition of New Quinoa Genotype, Grown in Hyper-Arid Desert Environments. Front. Plant Sci..

[42] Bradford M.M. (1976). A rapid and sensitive method for the quantitation of microgram quantities of protein utilizing the principle of protein-dye binding. Anal. Biochem..

[43] Kazerooni E.A., Al-Sadi A.M., Kim I.-D., Imran M., Lee I.-J. (2021). Ampelopsin Confers Endurance and Rehabilitation Mechanisms in Glycine max cv. Sowonkong under Multiple Abiotic Stresses. Int. J. Mol. Sci..

[44] Zhu J., Qi J., Fang Y., Xiao X., Li J., Lan J., Tang C. (2018). Characterization of Sugar Contents and Sucrose Metabolizing Enzymes in Developing Leaves of Hevea brasiliensis. Front. Plant Sci..

[45] Du Y., Zhao Q., Chen L., Yao X., Xie F.J.A. (2020). Effect of drought stress at reproductive stages on growth and nitrogen metabolism in soybean. Agronomy.

[46] Soffan A., Alghamdi S., Aldawood A.S. (2014). Peroxidase and polyphenol oxidase activity in moderate resistant and susceptible Vicia faba induced by *Aphis craccivora* (Hemiptera: Aphididae) infestation. J. Insect Sci..

[47] Zhou Y., Tang N., Huang L., Zhao Y., Tang X., Wang K. (2018). Effects of Salt Stress on Plant Growth, Antioxidant Capacity, Glandular Trichome Density, and Volatile Exudates of Schizonepeta tenuifolia Briq. Int. J. Mol. Sci..

[48] Batista-Silva W., Heinemann B., Rugen N., Nunes-Nesi A., Araújo W.L., Braun H.-P., Hildebrandt T.M. (2019). The role of amino acid metabolism during abiotic stress release. Plant Cell Environ..

[49] Choi W.-G., Toyota M., Kim S.-H., Hilleary R., Gilroy S. (2014). Salt stress-induced Ca^2+^ waves are associated with rapid, long-distance root-to-shoot signaling in plants. Proc. Natl. Acad. Sci. USA.

[50] Augusto A.D.S., Sperança M.A., Andrade D.F., Pereira-Filho E.R. (2017). Nutrient and Contaminant Quantification in Solid and Liquid Food Samples Using Laser-Ablation Inductively Coupled Plasma-Mass Spectrometry (LA-ICP-MS): Discussion of Calibration Strategies. Food Anal. Methods.

[51] Oñate-Sánchez L., Vicente-Carbajosa J. (2008). DNA-free RNA isolation protocols for Arabidopsis thaliana, including seeds and siliques. BMC Res. Notes.

[52] Jiang F., Wang J.-Y., Jia H.-F., Jia W.-S., Wang H.-Q., Xiao M. (2013). RNAi-mediated silencing of the flavanone 3-hydroxylase gene and its effect on flavonoid biosynthesis in strawberry fruit. J. Plant Growth Regul..

[53] Meng C., Zhang S., Deng Y.-S., Wang G.-D., Kong F.-Y. (2015). Overexpression of a tomato flavanone 3-hydroxylase-like protein gene improves chilling tolerance in tobacco. Plant Physiol. Biochem..

[54] Ji K., Kai W., Zhao B., Sun Y., Yuan B., Dai S., Li Q., Chen P., Wang Y., Pei Y. (2014). SlNCED1 and SlCYP707A2: Key genes involved in ABA metabolism during tomato fruit ripening. J. Exp. Bot..

[55] Zhu M., Chen G., Dong T., Wang L., Zhang J., Zhao Z., Hu Z. (2015). SlDEAD31, a putative DEAD-box RNA helicase gene, regulates salt and drought tolerance and stress-related genes in tomato. PLoS ONE.

[56] Habib S., Lwin Y.Y., Li N. (2021). Down-Regulation of SlGRAS10 in Tomato Confers Abiotic Stress Tolerance. Genes.

[57] Baron N.C., Rigobelo E.C. (2022). Endophytic fungi: A tool for plant growth promotion and sustainable agriculture. Mycology.

[58] Liu Y., von Wirén N. (2017). Ammonium as a signal for physiological and morphological responses in plants. J. Exp. Bot..

[59] Vatter T., Neuhäuser B., Stetter M., Ludewig U. (2015). Regulation of length and density of Arabidopsis root hairs by ammonium and nitrate. J. Plant Res..

[60] Sun P.-F., Fang W.-T., Shin L.-Y., Wei J.-Y., Fu S.-F., Chou J.-Y. (2014). Indole-3-acetic acid-producing yeasts in the phyllosphere of the carnivorous plant *Drosera indica* L.. PLoS ONE.

[61] Jiang S., Zhang D., Wang L., Pan J., Liu Y., Kong X., Zhou Y., Li D. (2013). A maize calcium-dependent protein kinase gene, ZmCPK4, positively regulated abscisic acid signaling and enhanced drought stress tolerance in transgenic Arabidopsis. Plant Physiol. Biochem..

[62] Harper J.F., Harmon A. (2005). Plants, symbiosis and parasites: A calcium signalling connection. Nat. Rev. Mol. Cell Biol..

[63] Ru J.-N., Hou Z.-H., Zheng L., Zhao Q., Wang F.-Z., Chen J., Zhou Y.-B., Chen M., Ma Y.-Z., Xi Y.-J. (2021). Genome-Wide Analysis of DEAD-box RNA Helicase Family in Wheat (*Triticum aestivum*) and Functional Identification of TaDEAD-box57 in Abiotic Stress Responses. Front. Plant Sci..

[64] Chen D., Wang Y., Zhang W., Li N., Dai B., Xie F., Sun Y., Sun M., Peng X. (2020). Gametophyte-specific DEAD-box RNA helicase 29 is required for functional maturation of male and female gametophytes in Arabidopsis. J. Exp. Bot..

[65] Yarra R., Xue Y. (2020). Ectopic expression of nucleolar DEAD-Box RNA helicase OsTOGR1 confers improved heat stress tolerance in transgenic Chinese cabbage. Plant. Cell Rep..

[66] Mittler R., Blumwald E. (2015). The roles of ROS and ABA in systemic acquired acclimation. Plant Cell.

[67] Huang Y., Guo Y., Liu Y., Zhang F., Wang Z., Wang H., Wang F., Li D., Mao D., Luan S. (2018). 9-cis-Epoxycarotenoid Dioxygenase 3 Regulates Plant Growth and Enhances Multi-Abiotic Stress Tolerance in Rice. Front. Plant Sci..

[68] Hauser F., Waadt R., Schroeder J.I. (2011). Evolution of Abscisic Acid Synthesis and Signaling Mechanisms. Curr. Biol..

[69] Dai S., Kai W., Liang B., Wang J., Jiang L., Du Y., Sun Y., Leng P. (2018). The functional analysis of SlNCED1 in tomato pollen development. Cell. Mol. Life Sci..

[70] Qin X., Zeevaart J.A.D. (1999). The 9-cis-epoxycarotenoid cleavage reaction is the key regulatory step of abscisic acid biosynthesis in water-stressed bean. Biol. Sci..

[71] Nambara E., Marion-Poll A. (2005). Abscisic acid biosynthesis and catabolism. Annu. Rev. Plant Biol..

[72] Muñoz-Espinoza V.A., López-Climent M.F., Casaretto J.A., Gómez-Cadenas A. (2015). Water Stress Responses of Tomato Mutants Impaired in Hormone Biosynthesis Reveal Abscisic Acid, Jasmonic Acid and Salicylic Acid Interactions. Front. Plant Sci..

[73] Fujita Y., Fujita M., Shinozaki K., Yamaguchi-Shinozaki K. (2011). ABA-mediated transcriptional regulation in response to osmotic stress in plants. J. Plant Res..

[74] Zhang D.-P., Zhou Y., Yin J.-F., Yan X.-J., Lin S., Xu W.-F., Baluška F., Wang Y.-P., Xia Y.-J., Liang G.-S. (2015). Rice G-protein subunits qPE9-1 and RGB1 play distinct roles in abscisic acid responses and drought adaptation. J. Exp. Bot..

[75] Bilal S., Shahzad R., Imran M., Jan R., Kim K.M., Lee I.-J. (2020). Synergistic association of endophytic fungi enhances Glycine max L. resilience to combined abiotic stresses: Heavy metals, high temperature and drought stress. Ind. Crops Prod..

[76] Hamayun M., Hussain A., Iqbal A., Khan S.A., Lee I.-J. (2018). Endophytic fungus Aspergillus japonicus mediates host plant growth under normal and heat stress conditions. BioMed Res. Int..

[77] Ramos P., Rivas N., Pollmann S., Casati P., Molina-Montenegro M.A. (2018). Hormonal and physiological changes driven by fungal endophytes increase Antarctic plant performance under UV-B radiation. Fungal Ecol..

[78] Rai M., Rathod D., Agarkar G., Dar M., Brestic M., Pastore G.M., Junior M.R.M. (2014). Fungal growth promotor endophytes: A pragmatic approach towards sustainable food and agriculture. Symbiosis.

[79] Wani A.B., Chadar H., Wani A.H., Singh S., Upadhyay N. (2016). Salicylic acid to decrease plant stress. Environ. Chem. Lett..

[80] Shaukat K., Zahra N., Hafeez M.B., Naseer R., Batool A., Batool H., Raza A., Wahid A., Aftab T., Naeem M. (2022). Chapter 2—Role of salicylic acid–induced abiotic stress tolerance and underlying mechanisms in plants. Emerging Plant Growth Regulators in Agriculture.

[81] Yuan S., Lin H.-H. (2008). Minireview: Role of salicylic acid in plant abiotic stress. Z. Nat. C.

[82] Nazar R., Umar S., Khan N.A. (2015). Exogenous salicylic acid improves photosynthesis and growth through increase in ascorbate-glutathione metabolism and S assimilation in mustard under salt stress. Plant Signal. Behav..

[83] Fayez K.A., Bazaid S.A. (2014). Improving drought and salinity tolerance in barley by application of salicylic acid and potassium nitrate. J. Saudi Soc. Agric. Sci..

[84] Zhang Y., Xu S., Yang S., Chen Y. (2015). Salicylic acid alleviates cadmium-induced inhibition of growth and photosynthesis through upregulating antioxidant defense system in two melon cultivars (*Cucumis melo* L.). Protoplasma.

[85] Kareem F., Rihan H., Fuller M.P. (2019). The effect of exogenous applications of salicylic acid on drought tolerance and up-regulation of the drought response regulon of Iraqi wheat. J. Crop Sci. Biotechnol..

[86] Miura K., Tada Y. (2014). Regulation of water, salinity, and cold stress responses by salicylic acid. Front. Plant Sci..

[87] Choudhury F.K., Rivero R.M., Blumwald E., Mittler R. (2017). Reactive oxygen species, abiotic stress and stress combination. Plant J..

[88] Mittler R., Vanderauwera S., Gollery M., Van Breusegem F. (2004). Reactive oxygen gene network of plants. Trends Plant Sci..

[89] Ahammed G.J., Wu M., Wang Y., Yan Y., Mao Q., Ren J., Ma R., Liu A., Chen S. (2020). Melatonin alleviates iron stress by improving iron homeostasis, antioxidant defense and secondary metabolism in cucumber. Sci. Hortic..

[90] Egbichi I., Keyster M., Jacobs A., Klein A., Ludidi N. (2013). Modulation of antioxidant enzyme activities and metabolites ratios by nitric oxide in short-term salt stressed soybean root nodules. S. Afr. J. Bot..

[91] Kissoudis C., Sunarti S., Van De Wiel C., Visser R.G., van der Linden C.G., Bai Y. (2016). Responses to combined abiotic and biotic stress in tomato are governed by stress intensity and resistance mechanism. J. Exp. Bot..

[92] Sachdev S., Ansari S.A., Ansari M.I., Fujita M., Hasanuzzaman M. (2021). Abiotic Stress and Reactive Oxygen Species: Generation, Signaling, and Defense Mechanisms. Antioxidants.

[93] Singh R.P., Jha P., Jha P.N. (2015). The plant-growth-promoting bacterium Klebsiella sp. SBP-8 confers induced systemic tolerance in wheat (Triticum aestivum) under salt stress. J. Plant Physiol..

[94] Batool T., Ali S., Seleiman M.F., Naveed N.H., Ali A., Ahmed K., Abid M., Rizwan M., Shahid M.R., Alotaibi M. (2020). Plant growth promoting rhizobacteria alleviates drought stress in potato in response to suppressive oxidative stress and antioxidant enzymes activities. Sci. Rep..

[95] Banik P., Zeng W., Tai H., Bizimungu B., Tanino K. (2016). Effects of drought acclimation on drought stress resistance in potato (*Solanum tuberosum* L.) genotypes. Environ. Exp. Bot..

[96] Kang J.-Y., Choi H.-I., Im M.-Y., Kim S.Y. (2002). Arabidopsis basic leucine zipper proteins that mediate stress-responsive abscisic acid signaling. Plant Cell.

[97] Yu Y., Qian Y., Jiang M., Xu J., Yang J., Zhang T., Gou L., Pi E. (2020). Regulation Mechanisms of Plant Basic Leucine Zippers to Various Abiotic Stresses. Front. Plant Sci..

[98] Heinemann B., Hildebrandt T.M. (2021). The role of amino acid metabolism in signaling and metabolic adaptation to stress-induced energy deficiency in plants. J. Exp. Bot..

[99] Grimplet J., Agudelo-Romero P., Teixeira R.T., Martinez-Zapater J.M., Fortes A.M. (2016). Structural and functional analysis of the GRAS gene family in grapevine indicates a role of GRAS proteins in the control of development and stress responses. Front. Plant Sci..

[100] Peng J., Carol P., Richards D.E., King K.E., Cowling R.J., Murphy G.P., Harberd N.P. (1997). The Arabidopsis GAI gene defines a signaling pathway that negatively regulates gibberellin responses. Genes Dev..

[101] Bolle C., Koncz C., Chua N.-H. (2000). PAT1, a new member of the GRAS family, is involved in phytochrome A signal transduction. Genes Dev..

[102] Helariutta Y., Fukaki H., Wysocka-Diller J., Nakajima K., Jung J., Sena G., Hauser M.-T., Benfey P.N. (2000). The SHORT-ROOT gene controls radial patterning of the Arabidopsis root through radial signaling. Cell.

[103] Stuurman J., Jäggi F., Kuhlemeier C. (2002). Shoot meristem maintenance is controlled by a GRAS-gene mediated signal from differentiating cells. Genes Dev..

[104] Mayrose M., Ekengren S.K., Melech-Bonfil S., Martin G.B., Sessa G. (2006). A novel link between tomato GRAS genes, plant disease resistance and mechanical stress response. Mol. Plant Pathol..

[105] Ma H.-S., Liang D., Shuai P., Xia X.-L., Yin W.-L. (2010). The salt-and drought-inducible poplar GRAS protein SCL7 confers salt and drought tolerance in Arabidopsis thaliana. J. Exp. Bot..

[106] Cui H. (2012). Killing two birds with one stone: Transcriptional regulators coordinate development and stress responses in plants. Plant Signal. Behav..

[107] Pandey S., Fartyal D., Agarwal A., Shukla T., James D., Kaul T., Negi Y.K., Arora S., Reddy M.K. (2017). Abiotic Stress Tolerance in Plants: Myriad Roles of Ascorbate Peroxidase. Front. Plant Sci..

[108] Ahmad P., Jaleel C.A., Salem M.A., Nabi G., Sharma S. (2010). Roles of enzymatic and nonenzymatic antioxidants in plants during abiotic stress. Crit. Rev. Biotechnol..

[109] Caverzan A., Casassola A., Brammer S.P. (2016). Antioxidant responses of wheat plants under stress. Genet. Mol. Biol..

[110] Sarkar S., Dey A., Kumar V., Batiha G.E.-S., El-Esawi M.A., Tomczyk M., Ray P. (2021). Fungal endophyte: An interactive endosymbiont with the capability of modulating host physiology in myriad ways. Front. Plant Sci..

[111] Jaakola L., Hohtola A. (2010). Effect of latitude on flavonoid biosynthesis in plants. Plant Cell Environ..

[112] Song X., Diao J., Ji J., Wang G., Guan C., Jin C., Wang Y. (2016). Molecular cloning and identification of a flavanone 3-hydroxylase gene from Lycium chinense, and its overexpression enhances drought stress in tobacco. Plant Physiol. Biochem..

[113] Mahajan M., Yadav S.K. (2014). Overexpression of a tea flavanone 3-hydroxylase gene confers tolerance to salt stress and Alternaria solani in transgenic tobacco. Plant Mol. Biol..

[114] Chutipaijit S., Cha-Um S., Sompornpailin K. (2009). Differential accumulations of proline and flavonoids in indica rice varieties against salinity. Pak. J. Bot..

[115] Banerjee A., Roychoudhury A., Singh V.P., Singh S., Tripathi D.K., Prasad S.M., Bhardwaj R., Chauhan D.K. (2021). 5—Role of sugars in mediating abiotic stress tolerance in legumes. Abiotic Stress and Legumes.

[116] O’Hara L.E., Paul M.J., Wingler A. (2013). How do sugars regulate plant growth and development? New insight into the role of trehalose-6-phosphate. Mol. Plant.

[117] Sami F., Yusuf M., Faizan M., Faraz A., Hayat S. (2016). Role of sugars under abiotic stress. Plant Physiol. Biochem..

[118] Vergara C., Araujo K.E.C., Alves L.S., Souza S.R.D., Santos L.A., Santa-Catarina C., Silva K.D., Pereira G.M.D., Xavier G.R., Zilli J.É. (2018). Contribution of dark septate fungi to the nutrient uptake and growth of rice plants. Braz. J. Microbiol..

[119] Sánchez F.J., Manzanares M.A., de Andres E.F., Tenorio J.L., Ayerbe L. (1998). Turgor maintenance, osmotic adjustment and soluble sugar and proline accumulation in 49 pea cultivars in response to water stress. Field Crops Res..

[120] Murakeözy É.P., Nagy Z., Duhazé C., Bouchereau A., Tuba Z. (2003). Seasonal changes in the levels of compatible osmolytes in three halophytic species of inland saline vegetation in Hungary. J. Plant Physiol..

[121] Kishor K., Bilhan P., Suravajhala R., Guddimalli R., Marka N., Kavya Shridhar K., Divya D., Scinthia K., Divya K., Doma M. (2020). Lysine, lysine-rich, serine, and serine-rich proteins: Link between metabolism, development, and abiotic stress tolerance and the role of ncRNAs in their regulation. Front. Plant Sci..

[122] Hildebrandt T.M. (2018). Synthesis versus degradation: Directions of amino acid metabolism during Arabidopsis abiotic stress response. Plant Mol. Biol..

[123] D’Mello J.F. (2015). Amino Acids in Higher Plants.

[124] Song M., Li X., Saikkonen K., Li C., Nan Z. (2015). An asexual Epichloë endophyte enhances waterlogging tolerance of Hordeum brevisubulatum. Fungal Ecol..

[125] Nasir Khan M., Siddiqui M.H., Mohammad F., Naeem M., Khan M.M.A. (2009). Calcium chloride and gibberellic acid protect linseed (*Linum usitatissimum* L.) from NaCl stress by inducing antioxidative defence system and osmoprotectant accumulation. Acta Physiol. Plant..

[126] Dawood M.G., El-Awadi M.E. (2015). Alleviation of salinity stress on *Vicia faba* L. plants via seed priming with melatonin. Acta Biológica Colomb..

[127] Kim J., Liu Y., Zhang X., Zhao B., Childs K.L. (2016). Analysis of salt-induced physiological and proline changes in 46 switchgrass (*Panicum virgatum*) lines indicates multiple response modes. Plant Physiol. Biochem..

[128] Trotel P., Bouchereau A., Niogret M.F., Larher F. (1996). The fate of osmo-accumulated proline in leaf discs of Rape (Brassica napus L.) incubated in a medium of low osmolarity. Plant Sci..

[129] Nanjo T., Kobayashi M., Yoshiba Y., Sanada Y., Wada K., Tsukaya H., Kakubari Y., Yamaguchi-Shinozaki K., Shinozaki K. (1999). Biological functions of proline in morphogenesis and osmotolerance revealed in antisense transgenic Arabidopsis thaliana. Plant J. Cell Mol. Biol..

