# Supplementary material for: Actinomucor elegans and Podospora bulbillosa Positively Improves Endurance to Water Deficit and Salinity Stresses in Tomato Plants
Source: J Fungi (Basel). 2022 Jul 27;8(8):785. doi: 10.3390/jof8080785 (PMC9409863; doi:10.3390/jof8080785)
Supplement: Supplementary file 1 [file jof-08-00785-s001.zip › jof-1806936-supplementary.pdf]

Supplementary materials

***Actinomucor elegans* and *Podospora bulbillosa* positively improves endurance to water deficit and salinity stresses in tomato plants**

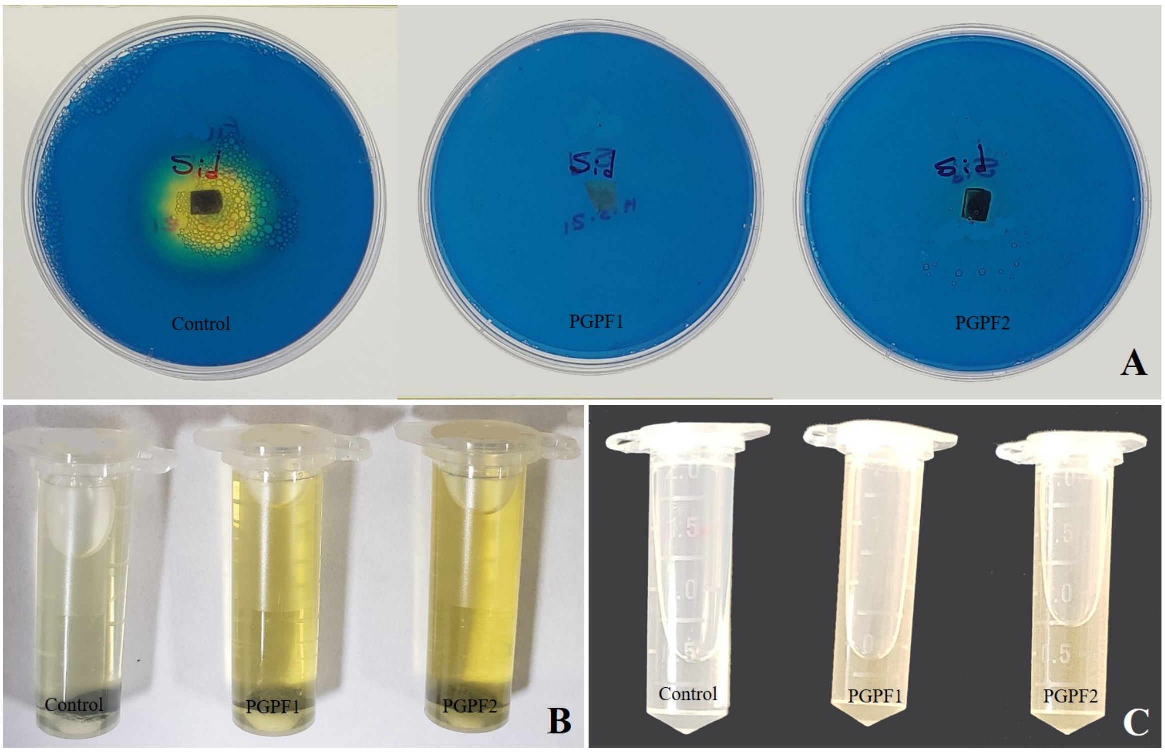

Figure S1. (A) Siderophore production, (B) Ammonia production, (C) IAA production,

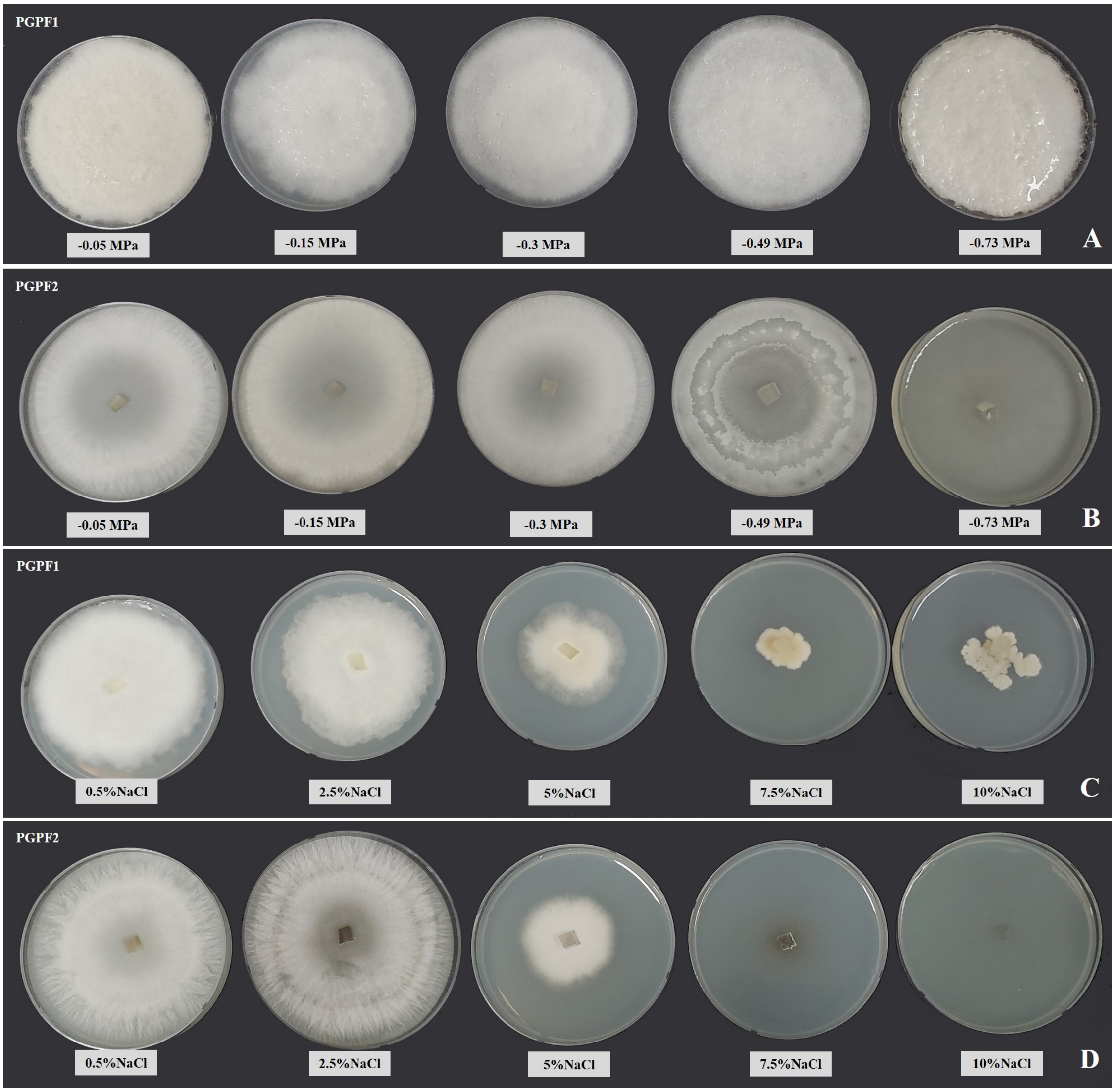

Figure S2. Drought (A and B) and Salt (C and D) tolerance ability of the selected fungal strains associated with *Solanum lycopersicum* in this study.

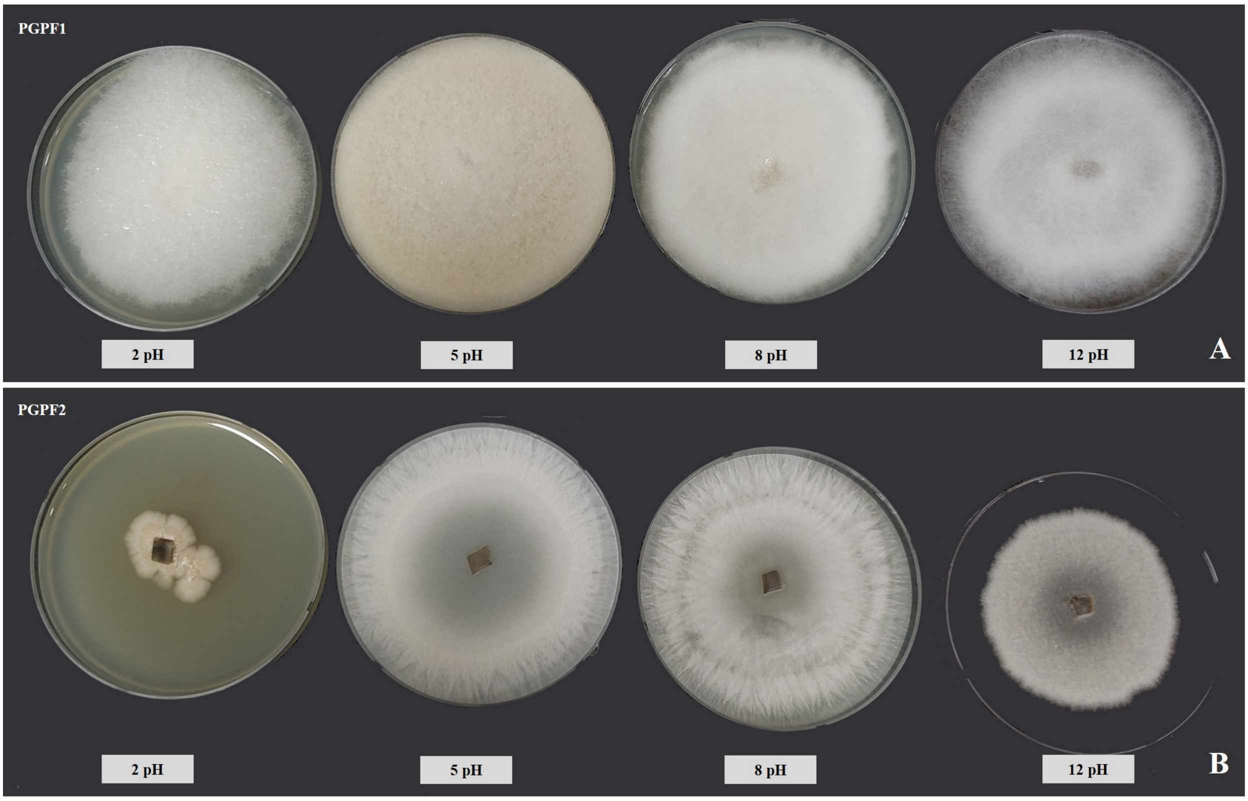

Figure S3. pH tolerance ability of the selected fungal strains associated with *Solanum lycopersicum* in this study (A,B).

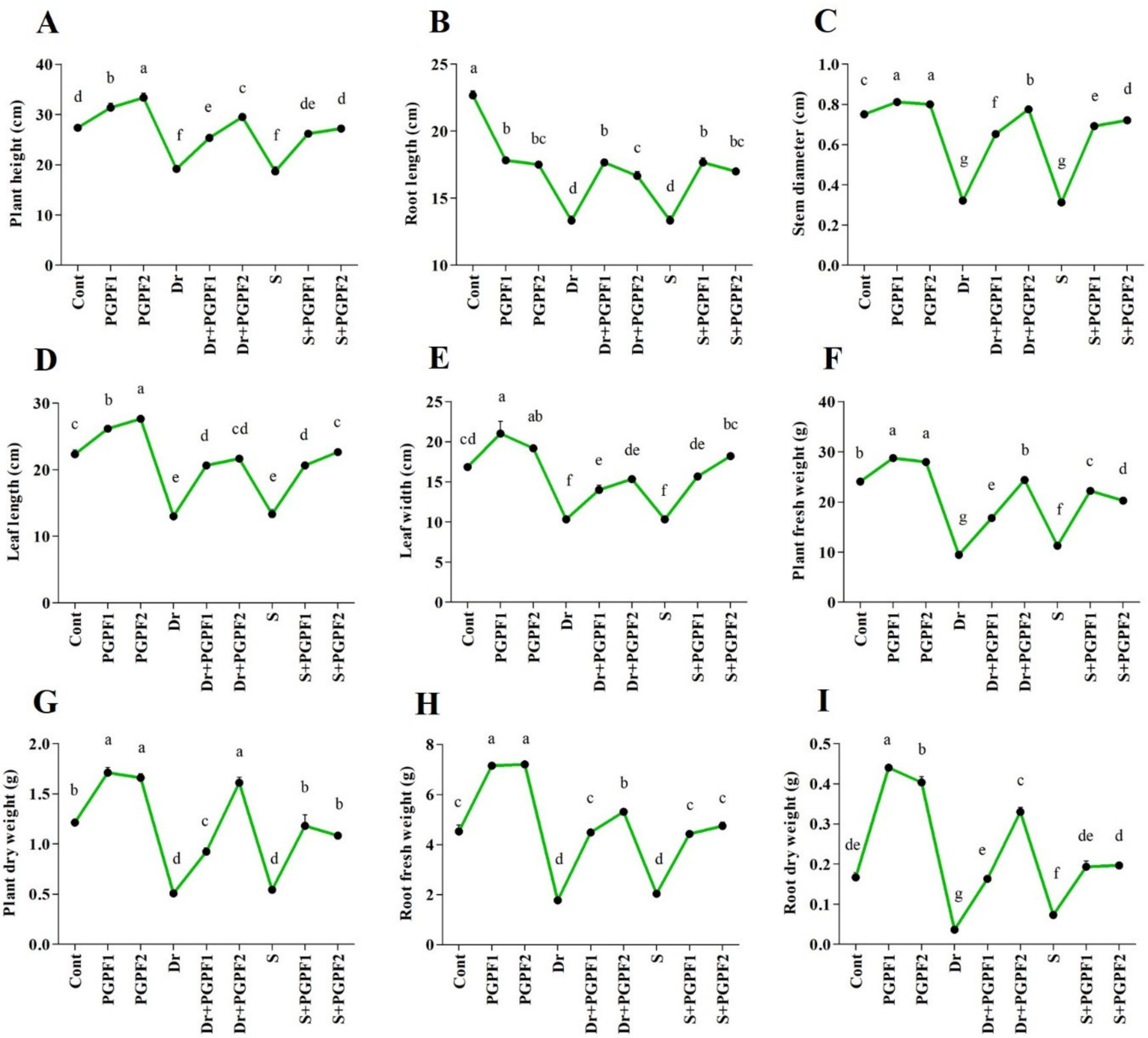

Figure S4. Effects of PGPF inoculation on tomato plant growth parameters under normal and stress conditions after 12 days of treatment (A-I). Treatments: Cont (control), PGPF1 (*Actinomucor elegans*), PGPF2 (*Podospora bulbillosa*), Dr (25% Polyethylene glycol), Dr (25% Polyethylene glycol) + PGPF1 (*Actinomucor elegans*), Dr (25% Polyethylene glycol) + PGPF2 (*Podospora bulbillosa*), S (1.5% sodium chloride), S (1.5% sodium chloride) + PGPF1 (*Actinomucor elegans*), and S (1.5% sodium chloride) + PGPF2 (*Podospora bulbillosa*). Values are shown as the means ± SD (n = 5) and significant differences at $p < 0.05$ (Tukey test) are indicated by different lowercase letters above the columns.

Table S1. Primers used for relative gene expression analysis.

Gene symbol	Primers (5′–3′) Forward/reverse
SlNCED1	5′-TCGAAAACCCGGATGAACAAGTGA-3′<br>5′-AACCAGAAACTTTTGGCCATGGTTC-3′
SlF3H	5′-GGGGACAAGTTTGTACAAAAAAGCAGGCTGATTCCATGGAAACCAAAGT-3′<br>5′-GGGGACCACTTTGTACAAGAAAGCTGGGTATCCCTATGAACCAAACGAC-3′
SlDEAD31	5′-GCTCTAGAAGTTACTGTCATAATAGAACCCTC-3′<br>5′-CGAGCTCGACAACAAAAACACAAATTCAT-3′
SlbZIP38	5′-CCATGCAAGCTTTCAAAGAAGCAGCTGT-3′<br>5′-GAGATGAATACGACGTACTAGAGTTGG-3′
SlGRAS10	5′-ACAGGGGATCGTAAACGGTG-3′<br>5′-ATTCATTGCCTCCCTGCCAA-3′
SlActin	5′-GGGATGGAGAAGTTTGGTGGTGG-3′<br>5′-CTTCGACCAAGGGATGGTGTAGC-3′
